# Increased Sampling and Intracomplex Homologies Favor Vertical Over Horizontal Inheritance of the Dam1 Complex

**DOI:** 10.1093/gbe/evad017

**Published:** 2023-02-15

**Authors:** Laura E van Rooijen, Eelco C Tromer, Jolien J E van Hooff, Geert J P L Kops, Berend Snel

**Affiliations:** Theoretical Biology and Bioinformatics, Department of Biology, Science Faculty, Utrecht University, Utrecht, The Netherlands; Cell Biochemistry, Groningen Biomolecular Sciences and Biotechnology Institute, Faculty of Science and Engineering, University of Groningen, Groningen, The Netherlands; Ecologie Systématique Evolution, CNRS, Université Paris-Saclay, AgroParisTech, Gif-sur-Yvette, France; Oncode Institute, Hubrecht Institute, Royal Netherlands Academy of Arts and Sciences, Utrecht, The Netherlands; Molecular Cancer Research, University Medical Center Utrecht, Utrecht, The Netherlands; Theoretical Biology and Bioinformatics, Department of Biology, Science Faculty, Utrecht University, Utrecht, The Netherlands

**Keywords:** kinetochore, protein complex evolution, intracomplex homologies, vertical inheritance, last eukaryotic common ancestor, horizontal gene transfer

## Abstract

Kinetochores connect chromosomes to spindle microtubules to ensure their correct segregation during cell division. Kinetochores of human and yeasts are largely homologous, their ability to track depolymerizing microtubules, however, is carried out by the nonhomologous complexes Ska1-C and Dam1-C, respectively. We previously reported the unique anti-correlating phylogenetic profiles of Dam1-C and Ska-C found among a wide variety of eukaryotes. Based on these profiles and the limited presence of Dam1-C, we speculated that horizontal gene transfer could have played a role in the evolutionary history of Dam1-C. Here, we present an expanded analysis of Dam1-C evolution, using additional genome as well as transcriptome sequences and recently published 3D structures. This analysis revealed a wider and more complete presence of Dam1-C in Cryptista, Rhizaria, Ichthyosporea, CRuMs, and Colponemidia. The fungal Dam1-C cryo-EM structure supports earlier hypothesized intracomplex homologies, which enables the reconstruction of rooted and unrooted phylogenies. The rooted tree of concatenated Dam1-C subunits is statistically consistent with the species tree of eukaryotes, suggesting that Dam1-C is ancient, and that the present-day phylogenetic distribution is best explained by multiple, independent losses and no horizontal gene transfer was involved. Furthermore, we investigated the ancient origin of Dam1-C via profile-versus-profile searches. Homology among 8 out of the 10 Dam1-C subunits suggests that the complex largely evolved from a single multimerizing subunit that diversified into a hetero-octameric core via stepwise subunit duplication and subfunctionalization of the subunits before the origin of the last eukaryotic common ancestor.

**Significance**

The Dam1 complex (Dam1-C) has a crucial role in eukaryotic cell division yet its distribution across species is very patchy. To resolve the evolutionary origin of this peculiar distribution, we used the recently acquired 3D structure to obtain a rooted phylogeny. This study makes an important step in discovering the evolutionary history of the Dam1-C, by determining that Dam1-C was part of the last eukaryotic common ancestor and arose via stepwise duplications during the transition from prokaryotes to eukaryotes.

## Introduction

During eukaryotic cell division, duplicate sister chromatids are equally divided by the microtubule-based spindle apparatus. Microtubules connect to chromatin via kinetochores, large protein structures that assemble onto centromeric DNA and that regulate equal separation into daughter cells (Cheeseman 2014). The final stages of chromosome segregation require kinetochores to hold on to depolymerizing microtubules. In yeasts, this role is performed by the Dam1 complex (Dam1-C), which interacts with the Ndc80 complex at kinetochores and forms a ring around the microtubules (Wang et al. 2007). In *Saccharomyces cerevisiae*, Dam1-C is essential and consists of 10 subunits: Dad1–Dad4, Dam1, Duo1, Ask1, Hsk3, Spc34, and Spc19 (Cheeseman, Brew, et al. 2001; Cheeseman, Enquist-Newman, et al. 2001; Enquist-Newman et al. 2001). In *Schizosaccharomyces pombe*, Dam1-C has the same function as in *S. cerevisiae*, but is not essential for short-term viability; nevertheless, mutations in the subunits do lead to increased chromosome mis-segregations (Thakur and Sanyal 2011).

Dam1-C has an enigmatic evolutionary history. It is the only complex of the yeast kinetochore that has a nonhomologous functional counterpart in humans. In humans and other animals, the role of Dam1-C is carried out by the nonhomologous three-subunit Ska complex (Ska-C; Cheeseman 2014). Orthologs of the subunits of both protein complexes occur across the eukaryotic tree of life largely in an anti-correlating fashion: 75 of the surveyed genomes contained one or more orthologs of the subunits of Dam1-C and Ska-C, and only seven genomes were predicted to contain orthologs of both complexes (Cipriano 2013; van Hooff et al. 2017). This striking alternating pattern could be indicative of a last eukaryotic common ancestor (LECA) possessing both Dam1-C and Ska-C, followed by independent, reciprocal loss. Alternatively, the pattern could have arisen through horizontal gene transfer (HGT) and displacement of one or both complexes across the eukaryotic tree of life. Phylogenetic analyses failed to unequivocally support either scenario, as critical branches in the phylogenies of the paralogs had low supports, and the phylogeny from the concatenated alignment of the full complex could not be rooted. HGT was ultimately favored over a LECA scenario, as HGT is “simpler”: the LECA scenario may imply both complexes to have had a different function in LECA in order to have co-existed, and to subsequently converge toward a kinetochore function, and, even more improbable, to do so in an alternating and independent manner in different lineages. In addition, it implies more losses and implies that many ancestral branches had both complexes, which contrasts the current-day underrepresentation of species with both.

**Fig. 1.**
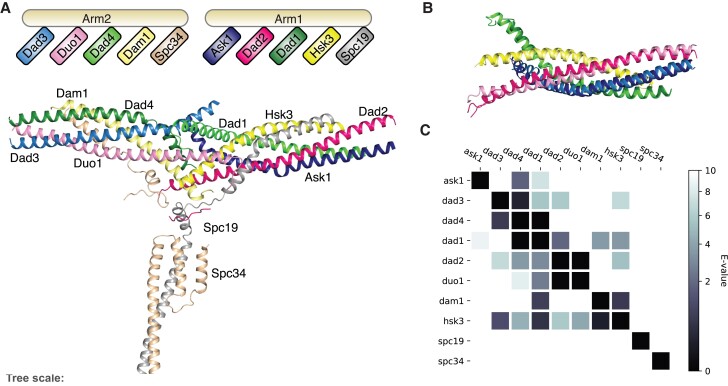
Dam1-C contains four paralogous pairs. (*A*) Structure of Dam1-C. The subunits are color coded according to the legend. (*B*) The structural alignment of Dam1-C subunits to their paralogs, and the subunits are color coded as in (*A*). (*C*) Heatmap of the profile-versus-profile hits for the separate subunits of the Dam1-C (*E* < 10).

A recent cryo-EM structure of the filamentous fungus *Chaetomium thermophilum* Dam1-C provided new insights (Jenni and Harrison 2018): This structure revealed that Dam1-C consists of two arms, each formed by five-helix bundles, with the N-termini of the subunits at the distal ends of the arms (fig. 1). From this structure, the authors perceived that each subunit has one “structural paralog” in the other arm, leading to five pairs: Ask1–Dad3, Dad2–Duo1, Dad4–Dad1, Hsk3–Dam1, and Spc19–Spc34. These putative paralogous relationships confirmed two previously inferred deep homologies based on profile-versus-profile comparisons (van Hooff et al. 2017) and the suggested three additional homologous pairs. Besides the implied large role for duplication in the history of the Dam1-C, the symmetrical structure of the Dam1-C in principle allows for a concatenation of the subunits of each separate arm and thereby infers a rooted tree—the root can be placed between the arms. Such a rooted phylogeny would enable to discern patterns of inheritance, including HGT, in contrast to the previously published, unrooted phylogeny based on an all-subunit concatenation.

Next to the 3D structure, new transcriptome and genome data are becoming available rapidly, and specifically relevant for DAM1-C are under-sampled groups such as CruMs, Cryptista, and Colponemidia. In addition to further uncovering the phylogenetic distribution of both complexes, these new data could aid in resolving their evolutionary histories. We utilize this novel sequence and structural information to improve our understanding of the evolutionary history of Dam1-C, including reconstructing a rooted phylogeny for Dam1-C. Our analyses suggest Dam1-C was already part of the LECA and indicate that this complex evolved through multiple intracomplex duplications.

## Results

### New Transcriptomes Reveal Near-Complete Dam1-C Presence in Multiple Lineages Outside Fungi

**Fig. 2.**
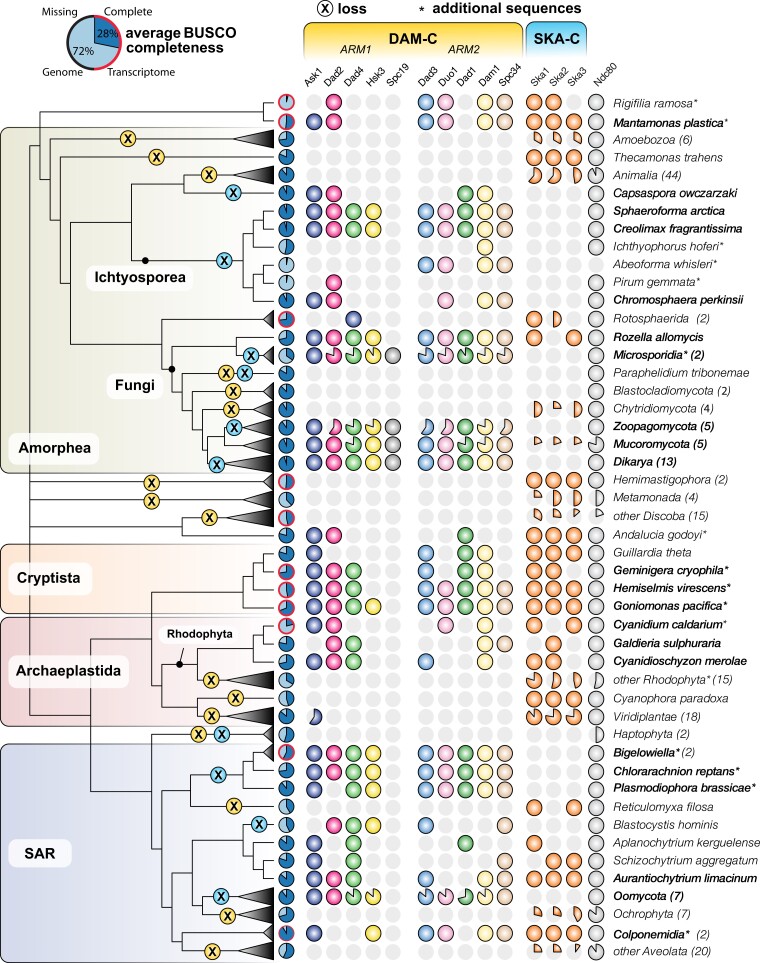
Presence-absence profiles of Dam1-C subunits, Ska-C, subunits and Ndc80 across selected eukaryotes. Filled circles denote presences. An asterisk indicates the presence of this species (strain) is a new addition compared with earlier work (van Hooff et al. 2017) and a red circle around the BUSCO chart indicates a transcriptome. The number behind each taxon describes the number of species in our genome/transcriptome set of that taxon. Pie charts indicate the percentage of species per taxon harbor a certain gene. Species in bold indicates the species is part of the tree in figure 3.

To revisit the evolutionary history of Dam1-C, a set of 181 eukaryotic species was compiled. This set expanded a previous data set in order to make use of predicted proteomes at phylogenetic positions that are relevant for Dam1-C and Ska-C (Deutekom et al. 2021) (fig. 2, supplementary fig. S1, Supplementary Material online). The most important additions were as follows: First, four Ichthyosporea were added to better investigate the presence of Dam1-C in deep-branching Holozoa (Grau-Bové et al. 2017). Second, early branching species within clades without known Dam1-C presence were added, including *Mantamonas plastica* and *Rigifila ramosa* (CRuMs), two Colponemidia (Alveolata), and *Andalucia godoyi* (Jakobida). Third, the data from the Marine Microbial Eukaryotic Transcriptome Sequence Project (MMETSP, version 3; Keeling et al. 2014; Johnson et al. 2019) were explored to find additional species having a near-complete presence of Dam1-C. In the MMETSP, three additional Rhizaria species were found with Dam1-C, and for *Bigelowiella natans*, a rhizarian, eight subunits instead of the previously detected five subunits were found in the strain designated CCMP1259. Fourth, three new *Cryptista* species were added to give strength to this group in addition to *Guillardia theta* from previous analyses, as *G. theta* is of interest because it has both Dam1-C and Ska-C. Fourth, an extensive search was performed to add more red algae (Rhodophyta) with Dam1-C to the data set, but only one additional species with Dam1-C subunits was found, namely *Cyanidium caldarium*. Finally, the genome of the filamentous fungus *C. thermophilum* was added, since this is the organism whose Dam1-C structure was elucidated (Jenni and Harrison 2018)

Orthologs of the 10 Dam1-C subunits were detected using profile Hidden Markov Model (HMM) searches against the compiled eukaryotic data set (see Methods). As expected, a complete Dam1-C complex was found among fungi (fig. 2). Dam1-C subunits remain undetected in Metazoa, but clear hits were observed in their unicellular relatives, namely the Ichtyosporea (e.g., *Abeoforma whisleri*, *Pirum gemmata*, *Ichthyophonus hoferi*, *Chromosphaera perkinsii*, *Creolimax fragrantissima*, and *Spaeroforma arctica*) and the filasterean *Capsaspora owczarzaki*. Orthologs of Dam1-C subunits were also detected in *Mantamonas*, which is currently thought to be sister to Amorphea (Fungi, Holozoa, Amoebozoa, and other unicellular relatives; Burki et al. 2020). Outside the Amorphea and CRuMs, a near-complete Dam1-C was found in Cryptista, Rhizaria, Stramenopila, and Jakobida. Five orthologous subunits of the Dam1-C were found in an early-diverging Alveolata branch (Colponemidia). Even though we found near-complete Dam1-C presences outside of Fungi, one Dam1-C subunit remained conspicuously absent in nonfungal lineages, namely Spc19, and we therefore deemed it fungi specific.

Genomes and transcriptomes in this study were selected for phylogenetic information and the best possible quality to obtain this information, but some sets of predicted proteins have far from perfect BUSCO scores (fig. 2). Incomplete subunit presence of Dam1-Cs especially in transcriptomes could therefore be attributed to incomplete data. However, this is likely not the dominant explanation as no difference was observed between the number of subunits found in transcriptomes and genomes (supplementary fig. S2, Supplementary Material online).

Comparing the phylogenetic profile of Ska-C (fig. 2) to that of Dam1-C using our expanded species set confirms the previous observations of a complementary phylogenetic distribution of Dam1-C and Ska-C (van Hooff et al. 2017). Most Ndc80-containing species (76%) have either Dam1-C (64/165) or Ska-C (92/165), and only 8% (14/165) of the species have both complexes. Dam1-C subunits were found in seven out of the nine eukaryotic major groups included in this study (CRuMs, Amorphea, Discoba, Metamonada, TSAR, Cryptista, and Archaeplastida), and Ska-C in eight (CRuMs, Amorphea, Discoba, Metamonada, Hemimastigophora, TSAR, Cryptista, and Archaeplastida). Thus, although the occurrence of Dam1-C is more widespread than previously estimated (e.g., present in CRuMs and early Colponemidia) and more complete in two lineages where it was observed before (from three to nine subunits in Ichthyosporea and from five to nine subunits in Cryptista), the tendency of Ska-C and Dam1-C to be mutually exclusive remains. However, Ska-Cs and Dam1-Cs combined presence in Alveolata and in a relative of fungi, Metazoa, and Amoebozoa (CRuMs) makes a stronger case for LECA carrying both Ska-C and Dam1-C. In addition, more sequences allowed for a renewed, richer phylogenetic investigation into their evolutionary histories.

### Four Paralogous Pairs in the Dam1-C

Jenni and Harrison (2018) suggested that Dam1-C consists of five “structural paralogs” (fig. 1*A*). Based on profile HMM comparisons, two sets of paralogous subunits were previously predicted in Dam1-C, namely Duo1–Dad2 and Dad1–Dad4–Ask1 (Brusini et al. 2022). Two of these paralogous pairs were confirmed by the structural paralogs: Duo1–Dad2 and Dad1–Dad4. To validate the proposed putative homologies, the two arms were structurally aligned (fig. 1*B*). The structures of the subunits Ask1–Dad3, Dad2–Duo1, Dad4–Dad1, and Hsk3–Dam1 can be superpositioned perfectly, as indicated by the low root mean square deviations (RMSD, Ask1–Dad3: 4.900 Å, Dad2–Duo1: 4.714 Å, Dad4–Dad1: 1.528 Å, and Hsk3–Dam1: 2.793 Å). Profile-versus-profile HMM searches between multiple alignments of orthologs of each Dam1-C subunit find as reciprocal best matches three of the four paralogous pairs as reciprocal best matches (fig. 1*C*, supplementary table S2, Supplementary Material online). Ask1–Dad3 is the exception, as these subunits are not directly retrieved in the profile-versus-profile HMM search, but they are linked via homology with other subunits (i.e., Dad3–Dad4 and Dad4–Ask1) and can be almost perfectly superpositioned as supported by the alignment of Ask1–Dad3 (RMSD: 4.900, fig. 1*B*) and structural all versus all comparison using Dali (supplementary fig. S3, Supplementary Material online; Holm 2020). The profile searches do not recover any homology between Spc19 and Spc34, which agrees with our inability to obtain significant structurally alignment of these subunits convincingly (RMSD: 27.318 Å). Our structural alignments combined with profile HMM-based sequence searches confirm that Dam1-C consists of four homologous pairs of paralogs.

### The Phylogenetic Tree of Concatenated Subunits of Both Dam1-C Arms is Consistent With the Species Tree

**Fig. 3.**
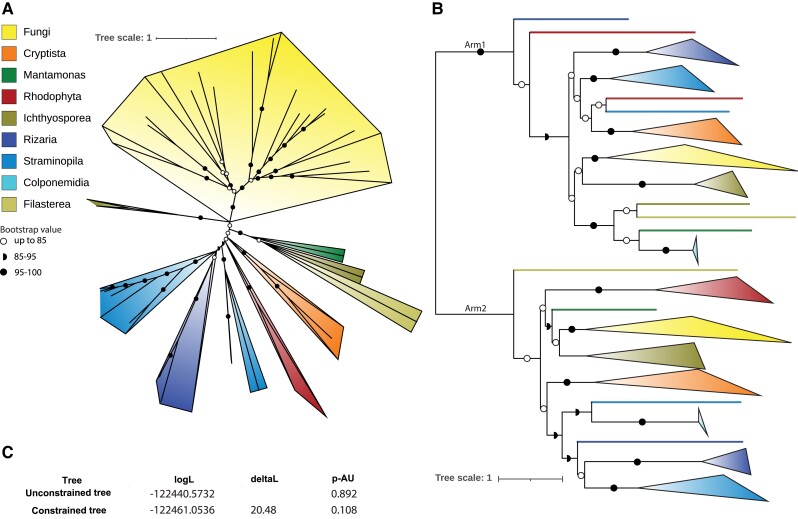
The phylogenetic tree of Dam1-C is consistent with the species tree. (*A*) Phylogeny inferred from concatenating the Dam1-C subunits, the eukaryotic groups are color coded as in the legend. (*B*) Phylogeny inferred from aligning the subunits pairs and concatenation of the subunits belonging to each arm. (*C*) Results from topology test (approximately unbiased, or AU test) of the rooted concatenated tree (‘Unconstrained tree”, the one depicted in (*B*)) and the constrained tree, which should follow the species tree. LogL is the likelihood of the specific tree. DeltaL is the difference between both trees and *P*-AU is the *P*-value derived from the topology test. A tree is rejected if *P* < 0.05.

Incongruence of a gene tree with the species tree is the gold standard to distinguish HGT from vertical descent. However, individual gene trees of Dam1-C subunits have a poor resolution due to limited phylogenetic signal in the (often) short sequences (supplementary fig. S4, Supplementary Material online). In contrast, the maximum likelihood phylogenetic tree based on a multiple alignment of concatenated Dam1-C is highly consistent with the eukaryotic phylogeny, indicating vertical descent (fig. 3*A*, supplementary fig. S5, Supplementary Material online). Nevertheless, depending on the placement of the root, it could also indicate an early HGT, for example, from fungi to other eukaryotic species. The symmetrical evolutionary history of the structure of Dam1-C allows for concatenation of the subunits from the two separate arms and thereby rooting of the resulting tree between the arms, to study vertical and horizontal signals in the evolution of Dam1-C. For a species to be suited for the rooted phylogenetic tree, we required it to have at least two subunits of the Dam1-C in both arms (fig. 2, selected species in bold). Spc19 and Spc34 were excluded from the concatenated alignment because structural alignments as well as profile-versus-profile searches did not unequivocally reveal homology between these two subunits and because Spc19 is lineage-specific in our homology searches.

The phylogeny inferred from the concatenation of the arms’ subunits yields a well-supported branch separating the two arms (fig. 3*B*, supplementary fig. S6, Supplementary Material online) and retrieves most of the major eukaryotic phyla/taxa as monophyletic clusters, albeit less consistently than the phylogeny from the full concatenation. The rooting by the arm-separating internal duplications allows us to see if any eukaryotic taxon could have been a donor of Dam1-C to all other lineages, which was previously hypothesized for fungi (van Hooff et al. 2017). In this specific hypothesis, one expects fungi to be located at the base of the phylogenetic tree, or in this case, of the clusters of each of the arms. In both the arm1 and arm2 clusters, fungi are not at the base, making it unlikely that Dam1-C originated in and was transferred from fungi. The most prominent incongruence with the species tree is the failure of *Plasmodiophora brassicae*'s failure to cluster with other Rhizaria. Inspection of the alignment revealed that the likely reason for this is incomplete data (gaps in the genes that are predicted) in *P. brassicae* and other Rhizaria.

Although both arms of the rooted tree are largely consistent with the species tree, they are not identical to the current consensus on the eukaryotic tree of life (Burki et al. 2020). To determine if the data are statistically significantly inconsistent with the consensus species tree, topology testing was used (approximately unbiased test, implemented in IQ-TREE (Shimodaira 2002; Nguyen et al. 2015)) was used. Specifically, we tested whether the tree obtained in figure 3*B* is significantly different from the species tree by constraining our phylogenetic analysis to obey the species tree (supplementary fig. S7, Supplementary Material online), and subsequently comparing the likelihoods of the tree in figure 3*B* and the constrained tree. The constrained trees are not significantly worse than the unconstrained tree (fig. 3*C*), revealing that a topology fully consistent with the species tree is an equally valid hypothesis for the evolution of Dam1-C. This supports vertical inheritance as a strong component of Dam1-C evolution.

The probability of HGT could also be informed by the localization of the different Dam1-C genes in the genome. After all, one would assume a single HGT event to have accounted for the transfer of all subunits at once. HGT of all subunits at once in turn probably would require the subunit genes to be localized in close proximity to one another. As a result, such an HGT event could leave a trace in current-day genomes in the form of subunit gene co-localization. However, in 18 out of the 32 species, the Dam1-C subunit genes were located on different scaffolds/chromosomes. Moreover, in the 13 where two or three of the subunits are on the same scaffold/chromosome, they were distantly located and on average ±560 kb nucleotides apart. Thus, Dam1-C subunits do not cluster in the current-day genomes, further undermining the probability of HGT of this complex.

### Homologies Within Dam1-C Arms Suggest a Large Role for Intracomplex Duplications in the Ancient Origin of Dam1-C

**Fig. 4.**
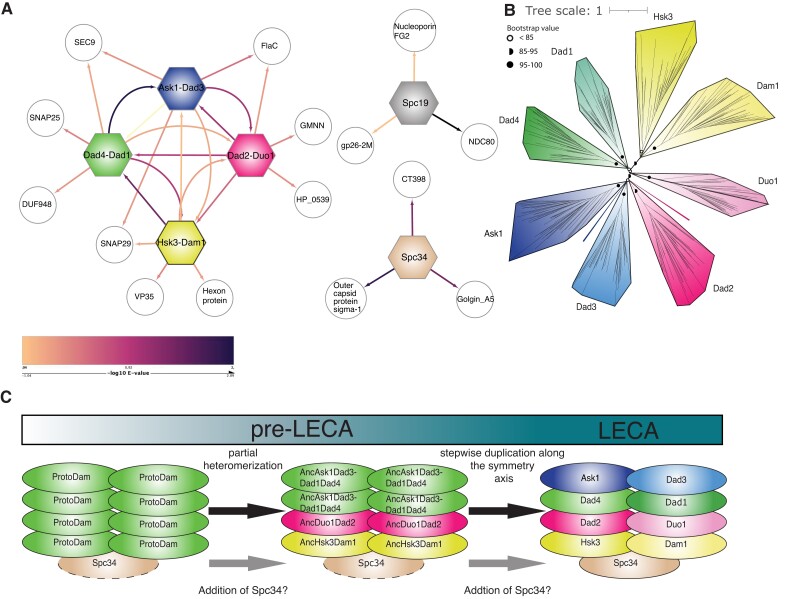
Relationships among the subunits and a scenario for the origin of Dam1-C. (*A*) The results of the profile-versus-profile HMM similarity searches are represented as a network. Hexanal nodes represent the profiles HMM of the merged paralogous subunits and the round nodes represent PDB and Pfam profiles. The lines indicate *E*-values, a darker line depicts a lower *E*-values. Eight Dam1-C subunits emerge as a consistent cluster. (*B*) Phylogeny of the eight paralogous Dam1-C subunits. Constructed using mafft merge and IQ-TREE, see Methods. (*C*) Scenario for the origin of the Dam1-C during the transition from prokaryotes to eukaryotes culminating in a nine subunit complex in LECA.

A pre-LECA origin of Dam1-C leaves open the question of how Dam1-C originated during the transition from prokaryotes to eukaryotes. Given that profile searches of individual Dam1-C subunits also hit other subunits than their closest homolog (fig. 1*C*), albeit at gray zone *E*-values, some of the established paralogous pairs could be homologous to one another as well. A profile-versus-profile HMM similarity search using merged alignments of the paralogous pairs indeed indicates additional homologous relations among the paralogous pairs (fig. 4*A*). The merged alignments of the paralogous pairs generally first hit the separate profiles of the separate subunits, then the profiles of the other paralogous pairs, and finally proteins outside of the complex. The putative deep homology between Dam1, Ask1, Dad1-4, Duo1, and Hsk3 allowed inferring a phylogeny containing these eight Dam1-C subunits (fig. 4*B*, supplementary fig. S8, Supplementary Material online). The clustering in this phylogeny confirms the closest paralogous relationships of Dam1–Hsk3, Dad1–Dad4, and Ask1–Dad3. Further inferences, however, are hampered by the low support values of the internal branches.

The homologies within the Dam1-C suggest a large role for intracomplex duplications in the origin of the Dam1-C. We suggest the following scenario based on the protein complex structure, the intracomplex homologies, and the phylogeny of subunits (fig. 4*C*). First, there was a single proto-Dam subunit that homo-multimerized into a Dam1-C like structure. Subsequent (stepwise) duplications resulted in the formation of a heteromer along the parallel axis (fig. 4*C*, step 2). The next wave of (stepwise) duplications occurred along the bifold symmetry axis. Subsequent subfunctionalization of the separate subunits resulted in an eight-subunit Dam1-C in early eukaryotic evolution. At some point before LECA, Spc34 was added. It could have been recruited when Dam1-C was still a homomer, or much later. Spc19 is most likely a (post-LECA) addition to the complex at the common ancestor of fungi.

## Discussion

Adding novel lineages (Colponemidia, Mantamonas) and increasing the resolution of previously analyzed lineages (Cryptophyta, Cercozoa) strengthens the unique phylogenetic distribution of Dam1-C as sparse yet wide, and largely anti-correlating with Ska-C. New structural data of Dam1-C (Jenni and Harrison 2018) confirmed previously postulated intracomplex homologies (van Hooff et al. 2017) and allowed inference of an arm-based phylogeny. These phylogenies strongly suggest that Dam1-C evolved through vertical descent, and the present-day genomic localization of the Dam1-C subunits is not consistent with enabling or receiving HGT. Altogether, this suggests that the complex was likely present in the LECA. Such an ancient origin for Dam1-C would mean that the sparse phylogenetic distribution is the result of widespread independent loss, having occurred in at least 14 lineages. This ancient origin also raises the question of the origin of the complex. Deep homologies inferred from merged profile-versus-profile HMM similarity searches and from the 3D structure suggest a scenario where Dam1-C arose through multiple rounds of intracomplex duplications, during eukaryogenesis.

An origin through intracomplex duplications during eukaryogenesis has been reconstructed for other complexes, providing precedent for such an inference in the case of Dam1-C. For example, the SM/LSM rings, which form a heteroheptameric ring, accompany the snRNA in the spliceosome. Each ring consists of seven proteins, which are all homologous and arose in two distinct waves of duplication. Between these waves of duplications, these copies underwent extensive sequence divergence, which makes determining the precise order of duplications difficult (Scofield and Lynch 2008; Veretnik et al. 2009).

Despite progress on elucidating the deep evolutionary history of Dam1-C, uncertainties remain. For example, it is not clear why the genomes of many organisms seem to contain only a subset of Dam1-C subunits. The inference of incomplete complexes could be due to data problems (see below) or to the difficulty to find homologs. Alternatively, Dam1-C subunits might be functional even if not all subunits are present, as large-scale studies of protein complexes have suggested (Fokkens and Snel 2009; Schultz and Seidl 2009) or the complex is functional by replacement of missing subunits (Morett et al. 2003). The uncertainty brought about by the partial presence of Dam1-C subunits is especially relevant in the Rhodophyta. Rhodophyta is one of the main lineages with a primary plastid that has spread by eukaryote-to-eukaryote endosymbiosis (Strassert et al. 2021). Although exhaustive searches were performed for Rhodophyta, the absence of any red alga with full Dam1-C prevents us to assess if part of the sparse distribution of Dam1-C can be partially explained by secondary endosymbiosis (re)introducing Dam1-C into lineages such as Stramenopila or Cryptophyta, both of which contain members that carry such a secondary plastid of red algal origin.

As the kinetochore is a fast-evolving complex, finding homologs has its difficulties (Hooff et al. 2017). A recent example is the kinetochore of Apicomplexa, where it is shown that subunits of the newly characterized kinetochore are distant homologs of canonical kinetochore proteins that were not identified in previous analyses (Brusini et al. 2022). To minimize the risk of missing subunits, sensitive methods were used, such as profile searches against a eukaryotic database. As the subunits of Dam1-C are shown to co-evolve, absences in species where other subunits were present, were treated as suspicious absences, and investigated manually. Even though we used sensitive methods to find the orthologs, additional subunits could be missed due to the difficulty to identify homologs or to potential data issues.

Although the arm-based phylogenetic tree is statistically consistent with the species tree (as described by Burki et al. (2020), more species and more sequences per species could solidify this result. Specifically, the paraphyly of Rhizara caused by the erroneous placement of *P. brassicae* is likely to be resolved when Dam1-C subunits from closely related species would be available. One of the potential data issues is contamination. We checked predicted Dam1-C subunit sequences for potential signatures of contamination and did not identify any. Even though we did not identify any putatively contaminated sequences, if species data sets were contaminated, the contamination must be relatively large to cause the spurious presence of multiple Dam1-C subunits. Another potential data issue is gene prediction problems. We relied on predicted proteomes and especially missing genes as well as wrongly predicted genes could have negatively impact phylogenetic resolution. The genes encoding Dam1-C subunits are short (average length: 102.8 amino acids) and therefore can be more easily missed by gene prediction software (Deutekom et al. 2019). We circumvented this issue by only including species with a minimum of two subunits per arm in the phylogenetic analysis.

Dam1-C joining Ska-C as present in early eukaryotic evolution eliminates the mystery of laterally transferring an entire complex among eukaryotes. Instead, it raises another issue; why would LECA have had two systems with the same function, and why were Dam1-C and Ska-C lost so often? Some intuition can come from other proteins with anti-correlating patterns, as these are not fully unique to Dam1-C and Ska-C. Two examples that also display such patterns and thus could help us to understand Dam1-C and Ska-C are (1) the paralogs elongation factor-I alpha (EF-1α) and elongation factor-like (EFL; Cocquyt et al. 2009; Kamikawa et al. 2013) and (2) the paralogs single subunit of ATP citrate lyase (ssACL) and double subunit ACL (Gawryluk et al. 2015). Both pairs were inferred to be present in LECA, and their anti-correlating pattern has been attributed to a combination of differential loss and specialization. The differential loss was speculated to stem from the overlapping function of the paralogs, which led one of the two proteins to become progressively less expressed, and subsequently lost in most existing lineages. In addition, especially in the case of EFL, the few species that do contain both proteins, one paralog is retained for a subset of the original functions and this subfunctionalization prevents losing this protein. These two examples are not fully comparable with Dam1-C and Ska1-C, since they pertain to paralogs instead of analogs and encompass single proteins instead of protein complexes; nevertheless, they provide support for functional redundancy at LECA followed by reciprocal loss and some degree of functional specialization to also have been at play for Dam1-C and Ska1-C.

Another explanation is that one of the complexes had a different function. It was recently shown that Dam1-C, in addition to its function in the kinetochore, also has a function in hyphal tip growth in fungi (Shah et al. 2019). This second function invites the hypothesis that in LECA, Ska-C had the kinetochore as the main function and Dam1-C had an additional function in another molecular process where microtubule tracking plays a role, like for example hyphal tip growth in some present-day fungi. Subsequently, loss of the need for this ancestral Dam1-C function in lineages such as Metazoa and Viridiplantae would incite the loss of Dam1-C, while a strong need for this ancestral Dam1-C function would allow the loss of Ska1-C, provided Dam1-C gained kinetochore activity. Under this scenario, it would not be deleterious to possess both Dam1-C and Ska-C (similar to EFL or ALC), and selection for gene loss from a feature-rich LECA explains the pattern. An additional two-function hypothesis is that the complexes were mitosis and meiosis specific, as is known for the paralogous cohesion subunits Rec8 and Rad21 (Parisi et al. 1999). In current-day species with both Dam1-C and Ska1-C, this would predict that either one of the complexes has a function in mitosis and the other in meiosis.

In summary, our phylogenomic findings propose that LECA contained both Dam1-C and Ska-C and that Dam1-C arose through intracomplex duplications. This hypothesis also raises questions as outlined above, which highlight the need for experimental cell biology to study the functional overlap and differentiation of these complexes in the—mostly understudied—organisms that contain both Dam1-C and Ska1-C, such as *Rhizophagus irregularis*, *G. theta*, or *Aurantiochytrium limacinum*.

## Methods

### Compiling the Proteome Database

For studying the presences and absences of subunits of Dam1-C, Ska-C, and Ndc80 across the eukaryotic tree of life, a data set was compiled containing the predicted proteomes, genomes, and transcriptomes from 181 eukaryotic organisms from different supergroups: 86 Opisthokonta, 6 Amoebozoa, 26 Archaeplastida, 4 Cryptista, 13 Excavata, 2 Haptophyta, 2 Hemimastigophora, 44 SAR, and 1 Apusozoa (supplementary table S2, Supplementary Material online). The initial set was constructed as described previously (Deutekom et al. 2021). This initial set was expanded using the following criteria: (1) species were selected to represent eukaryotic diversity and allow for a detailed analysis of the evolution of Dam1-C. (2) If available, two species were selected per clade, and commonly used model organisms were preferred over other species. (3) If multiple proteomes, genomes, or transcriptomes were available for a single species, the one with the most complete Dam1-C complex was selected.

The Cryptista and the extra Rhizaria were obtained from the MMETSP (version 3; Keeling et al. 2014; Johnson et al. 2019). To be able to use of the transcriptomes in MMETSP, we translated them using transeq (EMBOSS:6.6.0.0). We replaced *B. natans* strain CCMP2755 with strain CCMP1259 compared with van Hooff et al, because for this strain, we were able to find eight subunits instead of five subunits. For the Colponemidia, Mantamonas, Ichthyosporea, and Rhodophyta, the EukProt database version 2 was used (Richter et al. 2020).

### Orthologs Detection

Orthologs of Dam1-C, Ska-C, and Ndc80 (fig. 1) were obtained by use of profile HMM as constructed by van Hooff et al. (2017). Although profile HMM similarity searches are primarily homology searches, we have previously demonstrated that profile HMM models capture a single orthologous subunit per species for Dam1-C subunits. The “hmmsearch” tool from the HMMER package (http://hmmer.org/, HMMER 3.1b1) and the initial profiles from van Hooff et al. were used to search through the updated database to find the subunits in the newly added species. The profiles were updated by adding the subunits from the new species to the multiple sequence alignment (MSA) using MAFFT E-INS-i (MAFFT v7.271) and then were created using the “hmmbuild” tool from the HMMER package. When searching with these updated profiles, we did not detect any additional Dam1-C subunit orthologs. Due to the structure of the Dam1-C subunits, that is, coiled-coil structure, the HMMER output was assessed manually. If multiple hits per species were found, phylogenetic analysis was used to differentiate orthologs from paralogs. From the MMETSP data set, species were added if specific Dam1-C sequences in a species could be found (*E* < 10^−3^) and if it had four or more subunits after manual curation.

### Profile-versus-profile Searches

To investigate if all the subunits are homologous to one another, a profile-versus-profile search was performed (fig. 4*A*), using the database of Pfam 31, pdb70, a database of profiles of kinetochore proteins and of the merged alignments of the subunit pairs of the Dam1-C. The merged alignments were aligned using MAFFT merge E-INS-i (MAFFT v7.271) and filtered using trimAl with parameter gt = 0.05. The alignments were manually curated for gene prediction problems. HHsuite (version 3.3.0, tool hhsearch) was used for profile-versus-profile search using the merged protein alignments of the coupled paralogous subunits and the separate protein alignments for each subunit. The FASTA files used to create the merged profiles can be found in the supplementary files.

### Phylogenetic Analysis

For the subunit tree (fig. 4*B*), the paralogy of the Dam1-C subunits was confirmed by the profile-versus-profile results. The sequences of each orthologous group were aligned using MAFFT E-INS-I (Katoh et al. 2005; MAFFT v7.271). For inferring building the tree, we then aligned the MSAs of the subunits of Dam1-C by using MAFFT merge E-INS-i. The alignments are trimmed using trimAl (Capella-Gutiérrez et al. 2009; trimAl v1.4.rev15) with gt 0.05. IQ-TREE was used to select a substitution model and to infer the phylogeny as advised by Kalyaanamoorthy et al. (2017). IQ-TREE was run with a 1,000 ultrafast bootstraps, and the model selected by ModelFinder was VT-F-R7. The sequences of the orthologous groups can be found in supplementary files, as well as the alignments and the IQ-TREE tree file.

### Concatenated Tree

For the concatenated tree based on the arms in the structure of Dam1-C, the subunits were aligned to their structural paralogs (fig. 3*B*). These alignments were concatenated to create a phylogeny. Not all species have all the subunits of Dam1-C. Hence, to avoid noise due to lack of information, only if a species had two or more subunits in each arm, its subunit sequences were added to the concatenated alignment. The sequences of the subunits were aligned using MAFFT E-INS-i (MAFFT v7.271). To align the structural paralogs of the MAFFT merge, E-INS-i was used. By using MAFFT merge, the sub-MSA is preserved. The MSA was filtered using trimAL gt 0.05 (trimAl v1.4.rev15). IQ-TREE was used as described above, and the model used was LG + F + R5.

For the tree based on the eight paralogous subunits (fig. 4*B*), all alignments of the subunits were concatenated and filtered as previously described, and IQ-TREE was run with ModelFinder and used the LG + F + R5 model.

### Topology Testing

To test whether the tree from the arm-based concatenated phylogenetic tree is any different from what we believe is the root of the eukaryotic tree, a topology test was performed. First, trees were inferred constraining them based on the split in the major supergroups. For the constraining of the tree, six representative species were chosen based on completeness and behavior in previous trees. *Plasmodiophora brassicae*, red algae, and *Ca. owczarzaki* were added to the constraints to ensure the backbone followed the species tree. The arms of the tree were simultaneously constrained (supplementary fig. S7, Supplementary Material online). IQ-TREE was used to do topology testing to assess whether the constrained trees have a significantly lower likelihood than the unconstrained tree. The model used was LG + F + R5.

## Supplementary Material

evad017_Supplementary_Data

## Data Availability

The data underlying sequences and alignments for this article are available in their respective online supplementary data, Supplementary Material online. Links to the publicly available genomes and proteomes utilized in this study can be found in supplementary table S2, Supplementary Material online in the Supplementary materials.
