# Correction to: Increased Sampling and Intracomplex Homologies Favor Vertical Over Horizontal Inheritance of the Dam1 Complex

**DOI:** 10.1093/gbe/evae072

**Published:** 2024-04-09

This is a correction to: Laura E van Rooijen, Eelco C Tromer, Jolien J E van Hooff, Geert J P L Kops, Berend Snel, Increased Sampling and Intracomplex Homologies Favor Vertical Over Horizontal Inheritance of the Dam1 Complex, *Genome Biology and Evolution*, Volume 15, Issue 3, March 2023, evad017, https://doi.org/10.1093/gbe/evad017.

The published version of this article has been updated to make supplementary data files available.

