# Supplementary material for: Increased Sampling and Intracomplex Homologies Favor Vertical Over Horizontal Inheritance of the Dam1 Complex
Source: Genome Biol Evol. 2023 Feb 15;15(3):evad017. doi: 10.1093/gbe/evad017 (PMC9998035; doi:10.1093/gbe/evad017)
Supplement: evad017_Supplementary_Data [file evad017_supplementary_data.zip › evad017_Supplementary_Data.docx]

Increased sampling and intra-complex homologies favor vertical over horizontal inheritance of the Dam1 complex

Authors: Laura E. van Rooijen^1^, Eelco C. Tromer^2^, Jolien J. E. van Hooff^3^, Geert J. P. L. Kops^4,5^, Berend Snel^1^

1 Theoretical Biology and Bioinformatics, Department of Biology, Science Faculty, Utrecht University, Utrecht, Netherlands

2 Cell Biochemistry, Groningen Biomolecular Sciences and Biotechnology Institute, Faculty of Science and Engineering, University of Groningen, Groningen, Netherlands

3 Ecologie Systématique Evolution, CNRS, Université Paris-Saclay, AgroParisTech, Gif-sur-Yvette, France

4 Oncode Institute, Hubrecht Institute, Royal Netherlands Academy of Arts and Sciences, Utrecht, Netherlands

5 University Medical Centre Utrecht, Utrecht, Netherlands

**Supplementary Material**

Supplementary Figure S1. Full presence-absence table of Dam1-C subunits, Ska-C subunits and Ndc80. Related to Figure 2. The presences of all subunits of Dam1-C (blue), Ska-C (orange) and Ndc80 (grey).


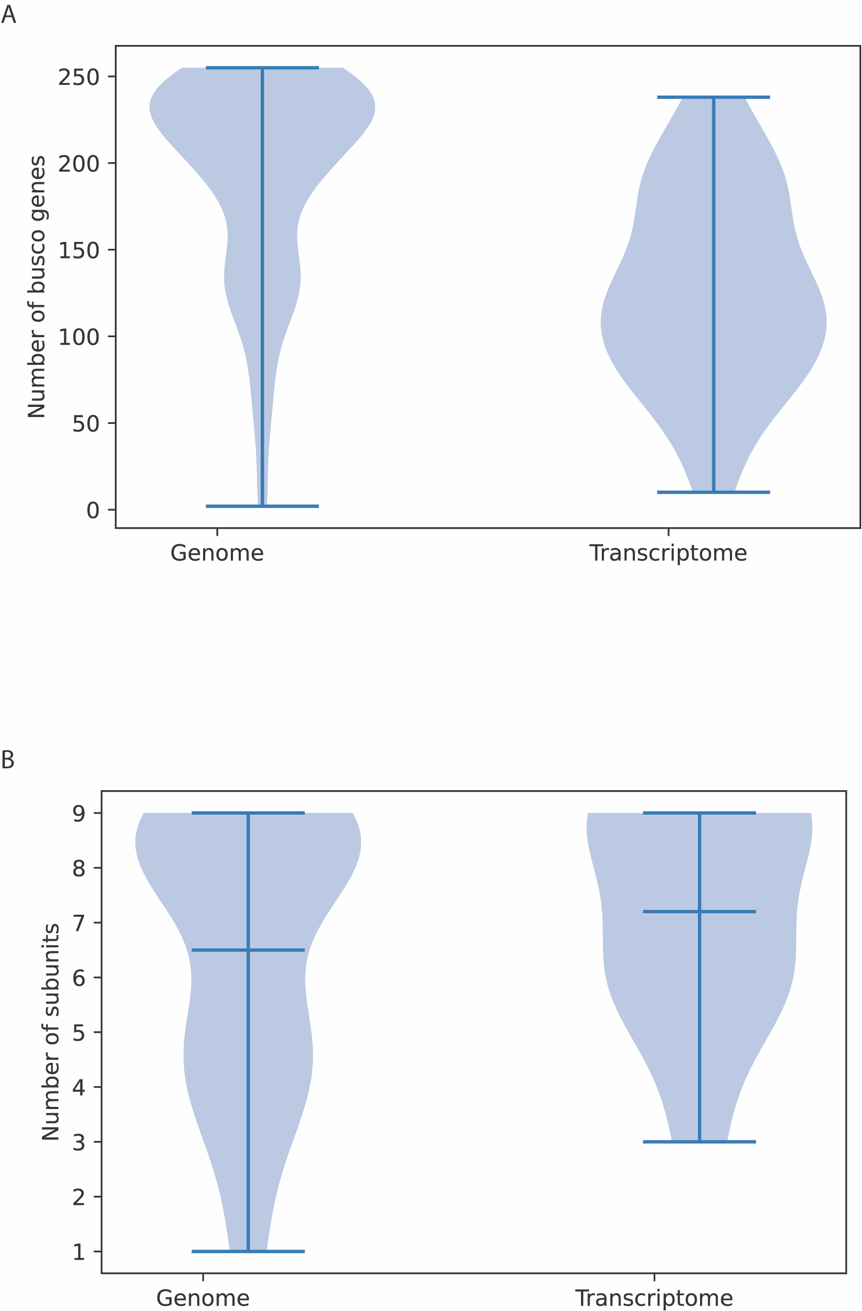


Supplementary Figure S2 A) violin plot of the BUSCO scores of all the transcriptomes and genomes. B)Violing plot of the number of subunits found when Dam1-C is present in genomes and transcriptomes

| Z-score | **Ask1** | **Dad3** | **Dam1** | **Hsk3** | **Dad1** | **Dad4** | **Dad2** | **Duo1** | **Spc19** | **Spc34** |
| --- | --- | --- | --- | --- | --- | --- | --- | --- | --- | --- |
| **Ask1** | 7.7 | 5.8 | 4.6 | 5.2 | 4.8 | 5.2 | 5.2 | 5.8 | 3.1 | 0.1 |
| **Dad3** | 5.8 | 7.9 | 4.8 | 5.3 | 5 | 5.2 | 5.1 | 5.6 | 3.6 | 0.1 |
| **Dam1** | 4.6 | 4.8 | 7.3 | 5.4 | 4.5 | 4.6 | 4.5 | 4.8 | 4.5 | 3.3 |
| **Hsk3** | 5.2 | 5.3 | 5.4 | 7.4 | 5.1 | 5.1 | 5 | 5.5 | 4.9 | 3.2 |
| **Dad1** | 4.8 | 5 | 4.5 | 5.1 | 7.2 | 4.8 | 4.8 | 5.2 | 4.4 | 2.4 |
| **Dad4** | 5.2 | 5.2 | 4.6 | 5.1 | 4.8 | 7.9 | 5.4 | 5.7 | 4.6 | 2.1 |
| **Dad2** | 5.2 | 5.1 | 4.5 | 5 | 4.8 | 5.4 | 8.3 | 6.2 | 4.7 | 0.1 |
| **Duo1** | 5.8 | 5.6 | 4.8 | 5.5 | 5.2 | 5.7 | 6.2 | 8.1 | 5.3 | 0.1 |
| **Spc19** | 3.1 | 3.6 | 4.5 | 4.9 | 4.4 | 4.6 | 4.7 | 5.3 | 8.4 | 2.6 |
| **Spc34** | 0.1 | 0.1 | 3.3 | 3.2 | 2.4 | 2.1 | 0.1 | 0.1 | 2.6 | 15.3 |

Supplementary Figure S3 Heatmap of structural similarity Z scores using Dali.


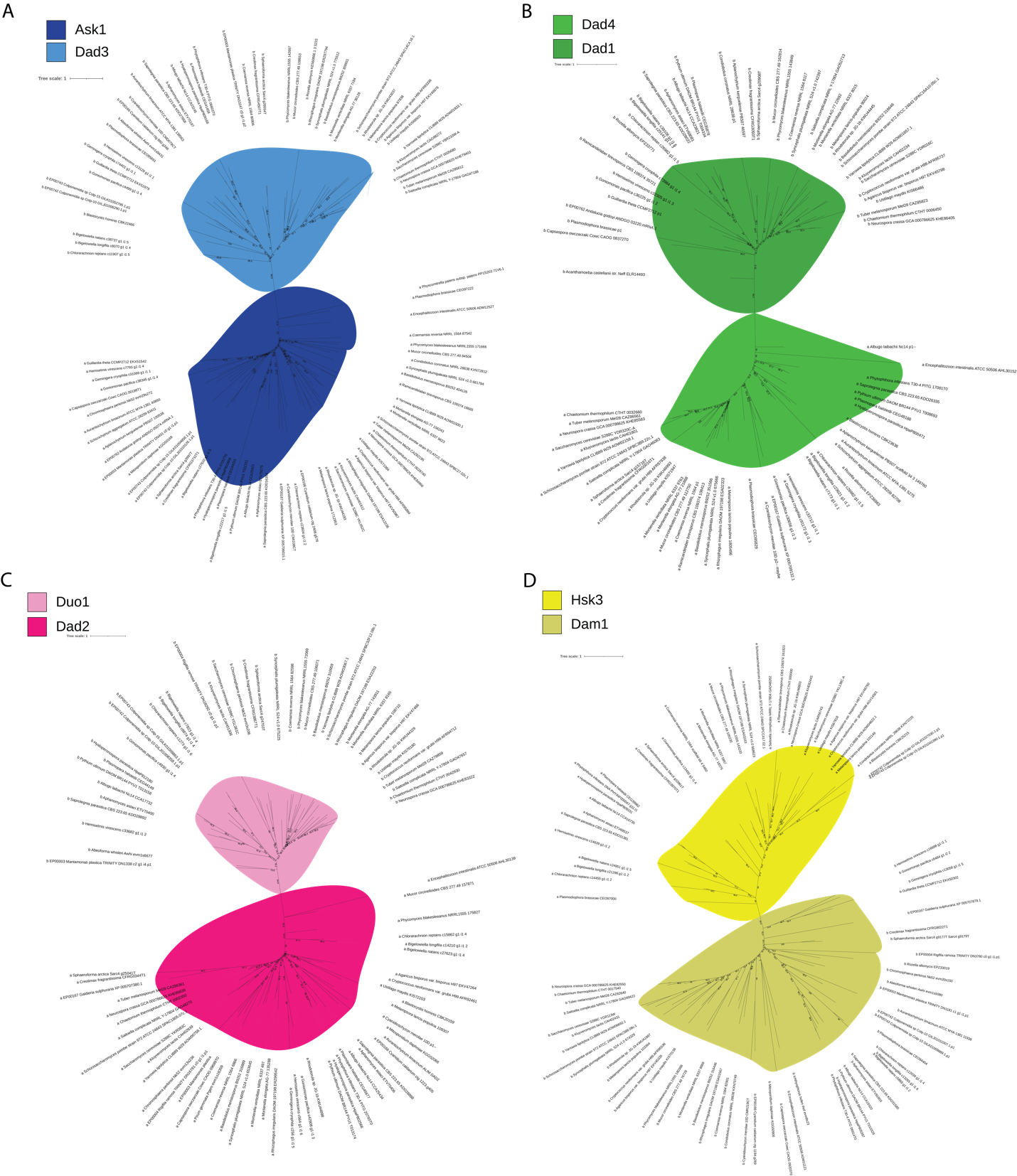
Supplementary Figure S4. Gene trees for Dam1-C paralog pairs. The leaves contain the identifiers of the protein sequences, the corresponding sequences can be found in the supplementary data A) Subunits tree of Ask1 (a) and Dad3 (b) Random seed number: 473931, Substitution model: VT+F+R4, B) Subunits tree Dad1 (a) and Dad4 (b) Random seed number: 804262 Substitution model: LG+R5. C) Subunit tree Duo1 (a) and Dad2 (b) Random seed number:87643, Substitution model VT+F+R4. D) Subunits tree Hsk3 (a) and Dam1 (b) Random seed number: 695565 Substitution model LG+F+R5.

Supplementary Figure S5 Concatenated tree of all Dam1-C subunits. Related to Figure 3. The leaf names are the species that these branches represent, i.e. whose subunits were part of the concatenated alignment. Random seed number: 74515, Model of substitution LG+F+R5.

Supplementary Figure S6 Phylogeny inferred from aligning the subunits pairs. Related to Figure 3. The leaves the species that these branches represent. Random seed number: 691067, Model of substitution LG+F+R5.

Supplementary Figure S7 The constrained phylogeny used for topology testing. Related to Figure 3. Random seed number: 99061, Model of substitution LG+F+R5.

Supplementary Figure S8 Phylogeny of all sequences of the eight paralogous Dam1-C subunits. Related to Figure 4. The leaves contain the species names and protein identifier. Random seed number: 333847, Model of substitution: VT+ R6

Supplementary Tabel S1. Table corresponding to the heatmap in Figure 1C, presenting the E-values of the profile vs profile searches. Rows are query and columns are target.

|  | **Ask1** | **Dad3** | **Dad4** | **Dad1** | **Dad2** | **Duo1** | **Dam1** | **Hsk3** | **Spc19** | **Spc34** |
| --- | --- | --- | --- | --- | --- | --- | --- | --- | --- | --- |
| **Ask1** | 1.50E-24 |  | 1.8 | 7.5 |  |  |  |  |  |  |
| **Dad3** |  | 3.00E-27 | 0.25 | 5.6 | 5.9 | 37 |  | 6.8 |  |  |
| **Dad4** |  | 0.71 | 4.10E-31 | 1.90E-17 | 18 | 21 | 10 | 31 |  |  |
| **Dad1** | 8.9 | 19 | 1.7E-16 | 6.6E-23 | 1.9 | 18 | 3.7 | 3.5 |  |  |
| **Dad2** |  | 6.9 | 3.6 | 3 | 2.90E-30 | 0.0035 | 17 | 5.3 |  |  |
| **Duo1** |  | 25 | 8.4 | 2.5 | 0.0033 | 3.70E-36 | 31 | 8.5 |  |  |
| **Dam1** |  |  |  | 0.79 |  |  | 1.20E-48 | 0.7 |  |  |
| **Hsk3** |  | 1.2 | 4.5 | 0.54 | 5.8 | 4.2 | 0.22 | 1.50E-22 |  |  |
| **Spc19** |  |  |  |  |  |  |  |  | 1.00E-33 |  |
| **Spc34** |  |  |  |  |  |  |  |  |  | 4.50E-61 |

Supplementary Table S2. Sources of proteomes used in this study

| Taxono-my ID | Scientific name | genome/  transcirptome | Data Source | Data Source Name | Download date |
| --- | --- | --- | --- | --- | --- |
| 5755 | Acanthamoeba castellanii str Neff | genome | ftp://ftp.ensemblgenomes.org/pub/protists/release-34/fasta/protists_amoebozoa1_collection/acanthamoeba_castellanii_str_neff/pep/Acanthamoeba_castellanii_str_neff.Acastellanii_strNEFF_v1.pep.all.fa.gz | Acastellanii_strNEFF_v1 | 20170124 |
| 5759 | Entamoeba histolytica | genome | ftp://ftp.ensemblgenomes.org/pub/protists/release-34/fasta/entamoeba_histolytica/pep/Entamoeba_histolytica.JCVI-ESG2-1.0.pep.all.fa.gz | JCVI-ESG2-1.0 | 20170124 |
| 370355 | Entamoeba invadens | genome | ftp://ftp.ensemblgenomes.org/pub/protists/release-34/fasta/protists_amoebozoa1_collection/entamoeba_invadens_ip1/pep/Entamoeba_invadens_ip1.EIA2_v2.pep.all.fa.gz | EIA2_v2 | 20170124 |
| 44689 | Dictyostelium discoideum | genome | ftp://ftp.ensemblgenomes.org/pub/protists/release-34/fasta/dictyostelium_discoideum/pep/Dictyostelium_discoideum.dicty_2.7.pep.all.fa.gz | dicty_2.7 | 20170124 |
| 13642 | Polysphondylium pallidum | genome | ftp://ftp.ensemblgenomes.org/pub/protists/release-34/fasta/protists_amoebozoa1_collection/polysphondylium_pallidum_pn500/pep/Polysphondylium_pallidum_pn500.PolPal_Dec2009.pep.all.fa.gz | PolPal_Dec2009 | 20170124 |
| 361139 | Acytostelium subglobosum | genome | ftp://ftp.ncbi.nlm.nih.gov/genomes/all/GCF/000/787/575/GCF_000787575.1_Asub_2.0/GCF_000787575.1_Asub_2.0_protein.faa.gz | Asub_2.0 | 20170124 |
| 3055 | Chlamydomonas reinhardtii | genome | ftp://ftp.ensemblgenomes.org/pub/plants/release-34/fasta/chlamydomonas_reinhardtii/pep/Chlamydomonas_reinhardtii.v3.1.pep.all.fa.gz | v3.1 | 20170124 |
| 145388 | Monoraphidium neglectum | genome | ftp://ftp.ncbi.nlm.nih.gov/genomes/all/GCF/000/611/645/GCF_000611645.1_mono_v1/GCF_000611645.1_mono_v1_protein.faa.gz | GCF_000611645.1 | 20170124 |
| 3067 | Volvox carteri | genome | http://genome.jgi.doe.gov/pages/dynamicOrganismDownload.jsf?organism=Phytozome | V2.1 | 20170124 |
| 3175 | Klebsormidium flaccidum | genome | http://genome.jgi.doe.gov/pages/dynamicOrganismDownload.jsf?organism=Phytozome | | 20170124 |
| 296587 | Micromonas species | genome | https://phytozome.jgi.doe.gov/biomart/martview/739c2860d5e06348b925fe0f410cb17c filename: Micromonas_species_RCC299_mart_export.txt | | 20170124 |
| 242159 | Ostreococcus lucimarinus | genome | ftp://ftp.ensemblgenomes.org/pub/plants/release-34/fasta/ostreococcus_lucimarinus/pep/Ostreococcus_lucimarinus.ASM9206v1.pep.all.fa.gz | ASM9206v1 | 20170124 |
| 554065 | Chlorella variabilis | genome | http://genome.jgi.doe.gov/ChlNC64A_1/download/Chlorella_NC64A.best_proteins.fasta.gz | | 20170117 |
| 248742 | Coccomyxa subellipsoidea | genome | https://phytozome.jgi.doe.gov/biomart/martview/739c2860d5e06348b925fe0f410cb17c filename: Coccomyxa_subellipsoidea_mart_export.txt | | 20170124 |
| 3329 | Picea abies | genome | ftp://plantgenie.org/Data/ConGenIE/Picea_abies/v1.0/FASTA/GenePrediction/Pabies1.01.0-HC-pep.faa.gz, ftp://plantgenie.org/Data/ConGenIE/Picea_abies/v1.0/FASTA/GenePrediction/Pabies1.01.0-MC-pep.faa.gz | v1.0 | 20170124 |
| 3197 | Marchantia polymorpha | genome | https://phytozome.jgi.doe.gov/biomart/martview/739c2860d5e06348b925fe0f410cb17c filename: Marchantia_polymorpha_mart_export.txt | | 20170124 |
| 3218 | Physcomitrella patens | genome | ftp://ftp.ensemblgenomes.org/pub/plants/release-34/fasta/physcomitrella_patens/pep/Physcomitrella_patens.ASM242v1.pep.all.fa.gz | ASM242v1 | 20170124 |
| 53036 | Sphagnum fallax | genome | https://phytozome.jgi.doe.gov/biomart/martview/739c2860d5e06348b925fe0f410cb17c filename: Sphagnum_fallax_mart_export.txt | | 20170124 |
| 4432 | Nelumbo nucifera | genome | ftp://ftp.ncbi.nlm.nih.gov/genomes/all/GCF/000/365/185/GCF_000365185.1_Chinese_Lotus_1.1/ | GCF_000365185.1 | 20170327 |
| 3702 | Arabidopsis thaliana | genome | ftp://ftp.ensemblgenomes.org/pub/plants/release-34/fasta/arabidopsis_thaliana/pep/Arabidopsis_thaliana.TAIR10.pep.all.fa.gz | TAIR10 | 20170124 |
| 218851 | Aquilegia coerulea Goldsmith | genome | https://phytozome.jgi.doe.gov/biomart/martview/739c2860d5e06348b925fe0f410cb17c filename: Aquilegia coerulea_mart_export.txt | | 20170124 |
| 13333 | Amborella trichopoda | genome | ftp://ftp.ensemblgenomes.org/pub/plants/release-34/fasta/amborella_trichopoda/pep/Amborella_trichopoda.AMTR1.0.pep.all.fa.gz | AMTR1.0 | 20170124 |
| 39947 | Oryza sativa japonica | genome | ftp://ftp.ensemblgenomes.org/pub/plants/release-34/fasta/oryza_sativa/pep/Oryza_sativa.IRGSP-1.0.pep.all.fa.gz | IRGSP-1.0 | 20170124 |
| 88036 | Selaginella moellendorffii | genome | https://phytozome.jgi.doe.gov/biomart/martview/739c2860d5e06348b925fe0f410cb17c filename: Selaginella_moellendorffii_mart_export.txt | v1.0 | 20170327 |
| 2762 | Cyanophora paradoxa | genome | http://cyanophora.rutgers.edu/cyanophora/Cyanophora_paradoxa_MAKER_gene_predictions-022111-aa.fasta | | 20170124 |
| 130081 | Galdieria sulphuraria | genome | ftp://ftp.ncbi.nlm.nih.gov/genomes/all/GCF/000/341/285/GCF_000341285.1_ASM34128v1/GCF_000341285.1_ASM34128v1_protein.faa.gz | | 20210527 |
| 45157 | Cyanidioschyzon merolae | genome | ftp://ftp.ensemblgenomes.org/pub/release-34/plants/fasta/cyanidioschyzon_merolae/pep/ | ASM9120v1 | 20170117 |
| 35688 | Porphyridium purpureum | genome | http://cyanophora.rutgers.edu/porphyridium/Porphyridium_genemodels_UPDATED.fasta | | 20170124 |
| 2769 | Chondrus crispus | genome | ftp://ftp.ensemblgenomes.org/pub/release-34/plants/fasta/plants_rhodophyta1_collection/chondrus_crispus/pep/ | ASM35022v2 | 20170117 |
| 2782 | Gracilariopsis lemaneiformis | genome | https://ftp.ncbi.nlm.nih.gov/genomes/all/GCA/003/346/895/GCA_003346895.1_Glem_v01/GCA_003346895.1_Glem_v01_genomic.fna.gz | v01 | 20210527 |
| 2771 | Cyanidium caldarium | genome | http://cyanophora.rutgers.edu/redEST/redESTs.tar.gz | Cyanidium.protein | 20210527 |
| 2787 | Porphyra purpurea | transcriptome | https://www.ncbi.nlm.nih.gov/sra/?term=txid2787[Organism:noexp] | Porphyra purpurea EST project | 20210527 |
| 2786 | Porphyra umbilicalis | genome | ftp://ftp.ncbi.nlm.nih.gov/genomes/all/GCA/002/049/455/GCA_002049455.2_P_umbilicalis_v1/GCA_002049455.2_P_umbilicalis_v1_protein.faa.gz | v1 | 20210527 |
| 2788 | Pyropia yezoensis | genome | http://nrifs.fra.affrc.go.jp/cgi-bin/lime_download/lime.cgi?nori_FASTA_AminoAcid | Ver. 1 | 20210527 |
| 48942 | Calliarthron tuberculosum | genome | http://realdb.algaegenome.org/P.c.t.html# | Calliarthron_tuberculosum_aa | 20210527 |
| 31481 | Hildenbrandia rubra | transcriptome | http://cyanophora.rutgers.edu/redEST/redESTs.tar.gz | est | 20210527 |
| 2822 | Palmaria palmata | transcriptome | http://cyanophora.rutgers.edu/redEST/redESTs.tar.gz | est | 20210527 |
| 291168 | Griffithsia okiensis | EST | https://www.ncbi.nlm.nih.gov/nuccore?LinkName=biosample_nuccore&from_uid=167350 | LIBEST_024364 | 20210527 |
| 28020 | Furcellaria lumbricalis | EST | https://www.ncbi.nlm.nih.gov/nuccore?LinkName=biosample_nuccore&from_uid=169272,https://www.ncbi.nlm.nih.gov/nuccore?LinkName=biosample_nuccore&from_uid=169271 | LIBEST_026199,LIBEST_026198 | 20210527 |
| 2769 | Chondrus crispus | genome | ftp://ftp.ncbi.nlm.nih.gov/genomes/all/GCF/000/350/225/GCF_000350225.1_ASM35022v2/GCF_000350225.1_ASM35022v2_protein.faa.gz | ASM35022v2 | 20210527 |
| 305493 | Eucheuma denticulatum | transcriptome | https://www.ncbi.nlm.nih.gov/sites/nuccore?term=379657[BioProject] | GFKZ01000000 | 20210527 |
| 172969 | Gracilaria changii | transcriptome | https://www.ncbi.nlm.nih.gov/sra?LinkName=biosample_sra&from_uid=7306656,https://www.ncbi.nlm.nih.gov/sra?LinkName=biosample_sra&from_uid=7306657 | GC_UT_1,GC_UT_2 | 20210527 |
| 753684 | Madagascaria erythrocladioides | transcriptome | https://doi.org/10.6084/m9.figshare.12410606 | MMETSP1450 | 20210527 |
| 867925 | Rhodochaete pulchella | transcriptome | http://cyanophora.rutgers.edu/redEST/redESTs.tar.gz | est | 20210527 |
| 31354 | Compsopogon caeruleus | transcriptome | https://doi.org/10.6084/m9.figshare.12410606 | MMETSP0312 | 20210527 |
| 1077150 | Erythrolobus australicus | transcriptome | https://doi.org/10.6084/m9.figshare.12410606 | MMETSP1353 | 20210527 |
| 708628 | Erythrolobus madagascarensis | transcriptome | https://doi.org/10.6084/m9.figshare.12410606 | MMETSP1354 | 20210527 |
| 2792 | Porphyridium aerugineum | transcriptome | https://doi.org/10.6084/m9.figshare.12410606 | MMETSP0313 | 20210527 |
| 35688 | Porphyridium purpureum | genome | http://porphyra.rutgers.edu/Porphyridium_purpureum_v2_genome_data.zip | v.2 | 20210527 |
| 708627 | Timspurckia oligopyrenoides | transcriptome | https://doi.org/10.6084/m9.figshare.12410606 | MMETSP1172 | 20210527 |
| 2801 | Rhodella violacea | transcriptome | https://doi.org/10.6084/m9.figshare.12410606 | MMETSP0167,MMETSP0314 | 20210527 |
| 282340 | Purpureofilum apyrenoidigerum | transcriptome | http://cyanophora.rutgers.edu/redEST/redESTs.tar.gz | est | 20210527 |
| 101924 | Rhodosorus marinus | transcriptome | https://doi.org/10.6084/m9.figshare.12410606 | MMETSP0011,MMETSP0315 | 20210527 |
| 446134 | Stylonematophyceae sp CCMP1999 | transcriptome | https://doi.org/10.6084/m9.figshare.12410606 | MMETSP1475 | 20210527 |
| 46947 | Geminigera cryophila | transcriptome | https://figshare.com/articles/Marine_Microbial_Eukaryotic_Transcriptome_Sequencing_Project_re-assemblies/3840153/3 | | 20201020 |
| 77927 | Hemiselmis virescens | transcirptome | https://figshare.com/articles/Marine_Microbial_Eukaryotic_Transcriptome_Sequencing_Project_re-assemblies/3840153/3 | | 20201020 |
| 55529 | Guillardia theta | genome | ftp://ftp.ensemblgenomes.org/pub/protists/release-34/fasta/guillardia_theta/pep/ | GCA_000315625.1 | 20170117 |
| 195067 | Goniomonas pacifica | transcriptome | https://figshare.com/articles/Marine_Microbial_Eukaryotic_Transcriptome_Sequencing_Project_re-assemblies/3840153/3 | | 20210527 |
| 856889 | Mantamonas plastica | transcriptome | https://trace.ncbi.nlm.nih.gov/Traces/sra/?run=SRR5997433 | Mantamonas plastica CCAP 1946/1 transcriptome | 20210527 |
| 1122280 | Rigifila ramosa | transcriptome | https://trace.ncbi.nlm.nih.gov/Traces/sra/?run=SRR5997435 | Rigifila ramosa CCAP 1967/1 transcriptome | 20210527 |
| 3039 | Euglena gracilis | transcriptome | ftp://ftp.pride.ebi.ac.uk/pride/data/archive/2019/01/PXD009998/Euglena_translated_transcriptome.fasta | translated_transcriptome | 20210527 |
| 3037 | Euglena longa | transcriptome | ftp://ftp.ncbi.nlm.nih.gov/sra/wgs_aux/GG/OE/GGOE01/GGOE01.1.fsa_nt.gz | GGOE01000000 | 20210527 |
| 38275 | Euglena mutabilis | transcriptome | https://trace.ncbi.nlm.nih.gov/Traces/sra/?run=ERR351290,https://trace.ncbi.nlm.nih.gov/Traces/sra/?run=ERR351289,https://trace.ncbi.nlm.nih.gov/Traces/sra/?run=ERR351288,https://trace.ncbi.nlm.nih.gov/Traces/sra/?run=ERR351287,https://trace.ncbi.nlm.nih.gov/Traces/sra/?run=ERR351286,https://trace.ncbi.nlm.nih.gov/Traces/sra/?run=ERR351285 | Population Genomics of Euglena mutabilis | 20210527 |
| 75058 | Bodo saltans | genome | ftp://ftp.ensemblgenomes.org/pub/release-34/protists/fasta/protists_euglenozoa1_collection/bodo_saltans/pep/ | | 20170105 |
| 1314962 | Perkinsela sp | genome | ftp://ftp.ensemblgenomes.org/pub/release-34/protists/fasta/protists_euglenozoa1_collection/perkinsela_sp_ccap_1560_4/pep/ | ASM123584v1 | 20170105 |
| 347515 | Leishmania major strain Friedlin | genome | ftp://ftp.ensemblgenomes.org/pub/release-34/protists/fasta/leishmania_major/pep/ | ASM272v2 | 20170105 |
| 5691 | Trypanosoma brucei | genome | ftp://ftp.ensemblgenomes.org/pub/release-34/protists/fasta/trypanosoma_brucei/pep/ | Chr11 | 20170105 |
| 59799 | Angomonas deanei | genome | ftp://ftp.ensemblgenomes.org/pub/release-34/protists/fasta/protists_euglenozoa1_collection/angomonas_deanei/pep/ | GCA_000442575.2 | 20170105 |
| 28005 | Strigomonas culicis | genome | ftp://ftp.ensemblgenomes.org/pub/release-34/protists/fasta/protists_euglenozoa1_collection/strigomonas_culicis/pep/ | GCA_000442495.1 | 20170105 |
| 134013 | Phytomonas sp | genome | ftp://ftp.ensemblgenomes.org/pub/release-34/protists/fasta/protists_euglenozoa1_collection/phytomonas_sp_isolate_hart1/pep/ | AKI_PRJEB1539_v1 | 20170105 |
| 5741 | Giardia intestinalis | genome | http://giardiadb.org/common/downloads/release-29/GintestinalisAssemblageAWB/fasta/data/ | GiardiaDB-29 Assemblage A isolate WB | 20170105 |
| 348837 | Spironucleus salmonicida | genome | ftp://ftp.ensemblgenomes.org/pub/release-34/protists/fasta/protists_fornicata1_collection/spironucleus_salmonicida/pep/ | SSK3.0 | 20170105 |
| 453998 | Monocercomonoides sp | genome | http://www.protistologie.cz/hampllab/data.html | Mono14B | 20170303 |
| 5722 | Trichomonas vaginalis | genome | ftp://ftp.ensemblgenomes.org/pub/release-34/protists/fasta/protists_parabasalia1_collection/trichomonas_vaginalis_g3/pep/ | G3 | 20170105 |
| 5762 | Naegleria gruberi | genome | ftp://ftp.ensemblgenomes.org/pub/release-34/protists/fasta/protists_heterolobosea1_collection/naegleria_gruberi/pep/ | v1 | 20170105 |
| 2903 | Emiliania huxleyi | genome | ftp://ftp.ensemblgenomes.org/pub/protists/release-34/fasta/emiliania_huxleyi/pep/ | GCA_000372725.1 | 20170117 |
| 1460289 | Chrysochromulina tobin strain | genome | ftp://ftp.ncbi.nlm.nih.gov/genomes/all/GCA/001/275/005/GCA_001275005.1_Ctobinv2/GCA_001275005.1_Ctobinv2_protein.faa.gz | GCA_001275005.1 v2 | 20170117 |
| 2027451 | Hemimastix kukwesjijk | transcriptome | https://datadryad.org/resource/doi:10.5061/dryad.n5g39d7/4 | assembly_July2016 | 20181121 |
| 2027454 | Spironema sp | transcriptome | https://datadryad.org/resource/doi:10.5061/dryad.n5g39d7/4 | assembly_July2016 | 20181121 |
| 7955 | Danio rerio | genome | ftp://ftp.ensembl.org/pub/release-87/fasta/danio_rerio/pep/Danio_rerio.GRCz10.pep.all.fa.gz | GRCz10 | 20170119 |
| 31033 | Takifugu rubripes | genome | ftp://ftp.ncbi.nlm.nih.gov/genomes/all/GCF/000/180/615/GCF_000180615.1_FUGU5/GCF_000180615.1_FUGU5_protein.faa.gz | FUGU5 | 20170119 |
| 8364 | Xenopus tropicalis | genome | ftp://ftp.ensembl.org/pub/release-87/fasta/xenopus_tropicalis/pep/ | Xenopus_tropicalis_JGI_4.2 | 20170119 |
| 294128 | Hyalella azteca | genome | ftp://ftp.ncbi.nlm.nih.gov/genomes/all/GCF/000/764/305/GCF_000764305.1_Hazt_2.0/GCF_000764305.1_Hazt_2.0_protein.faa.gz | Hazt_2.0 | 20170119 |
| 283909 | Capitella teleta | genome | ftp://ftp.ensemblgenomes.org/pub/metazoa/release-34/fasta/capitella_teleta/pep/Capitella_teleta.GCA_000328365.1.pep.all.fa.gz | Capitella teleta v1.0 | 20170119 |
| 407821 | Stegodyphus mimosarum | genome | ftp://ftp.ensemblgenomes.org/pub/metazoa/release-34/fasta/stegodyphus_mimosarum/pep/Stegodyphus_mimosarum.GCA_000611955.2.pep.all.fa.gz | Stegodyphus_mimosarum_v1 | 20170119 |
| 8839 | Anas platyrhynchos | genome | ftp://ftp.ensembl.org/pub/release-87/fasta/anas_platyrhynchos/pep/Anas_platyrhynchos.BGI_duck_1.0.pep.all.fa.gz | BGI_duck_1.0 | 20170119 |
| 29159 | Crassostrea gigas | genome | ftp://ftp.ensemblgenomes.org/pub/metazoa/release-34/fasta/crassostrea_gigas/pep/Crassostrea_gigas.GCA_000297895.1.pep.all.fa.gz | oyster_v9 | 20170119 |
| 6669 | Daphnia pulex | genome | ftp://ftp.ensemblgenomes.org/pub/metazoa/release-34/fasta/daphnia_pulex/pep/Daphnia_pulex.GCA_000187875.1.pep.all.fa.gz | V1.0 | 20170119 |
| 7739 | Branchiostoma floridae | genome | ftp://ftp.ncbi.nlm.nih.gov/genomes/all/GCA/000/003/815/GCA_000003815.1_Version_2/GCA_000003815.1_Version_2_protein.faa.gz | GCA_000003815.1_Version_2_genomic | 20170119 |
| 37653 | Octopus bimaculoides | genome | ftp://ftp.ensemblgenomes.org/pub/metazoa/release-34/fasta/octopus_bimaculoides/pep/Octopus_bimaculoides.PRJNA270931.pep.all.fa.gz | Octopus_bimaculoides_v2_0 | 20170119 |
| 7868 | Callorhinchus milii | genome | ftp://ftp.ncbi.nlm.nih.gov/genomes/all/GCF/000/165/045/GCF_000165045.1_Callorhinchus_milii-6.1.3/GCF_000165045.1_Callorhinchus_milii-6.1.3_protein.faa.gz | Callorhinchus_milii-6.1.3 | 20170119 |
| 70779 | Acropora digitifera | genome | ftp://ftp.ncbi.nlm.nih.gov/genomes/all/GCF/000/222/465/GCF_000222465.1_Adig_1.1/GCF_000222465.1_Adig_1.1_protein.faa.gz | Adig1.1 | 20170119 |
| 45351 | Nematostella vectensis | genome | ftp://ftp.ensemblgenomes.org/pub/metazoa/release-34/fasta/nematostella_vectensis/pep/Nematostella_vectensis.GCA_000209225.1.pep.all.fa.gz | ASM20922v1 - NemVe_1 | 20170119 |
| 6087 | Hydra vulgaris | genome | ftp://ftp.ncbi.nlm.nih.gov/genomes/all/GCF/000/004/095/GCF_000004095.1_Hydra_RP_1.0/GCF_000004095.1_Hydra_RP_1.0_protein.faa.gz | Hydra_RP-1.0 | 20170119 |
| 7070 | Tribolium castaneum | genome | ftp://ftp.ncbi.nlm.nih.gov/genomes/all/GCA/000/002/335/GCA_000002335.3_Tcas5.2/GCA_000002335.3_Tcas5.2_protein.faa.gz | Tcas5.2 | 20170119 |
| 7227 | Drosophila melanogaster | genome | ftp://ftp.ensemblgenomes.org/pub/metazoa/release-34/fasta/drosophila_melanogaster/pep/Drosophila_melanogaster.BDGP6.pep.all.fa.gz | BDGP6 | 20170119 |
| 7165 | Anopheles gambiae | genome | ftp://ftp.ensemblgenomes.org/pub/metazoa/release-34/fasta/anopheles_gambiae/pep/ | Agam4.4 | 20170119 |
| 7668 | Strongylocentrotus purpuratus | genome | http://www.echinobase.org/Echinobase/SpDownloads Version 4.2 | Spur4.2 | 20170119 |
| 225164 | Lottia gigantea | genome | ftp://ftp.ensemblgenomes.org/pub/metazoa/release-34/fasta/lottia_gigantea/pep/Lottia_gigantea.GCA_000327385.1.pep.all.fa.gz | Lotgi1 | 20170119 |
| 10224 | Saccoglossus kowalevskii | genome | ftp://ftp.ncbi.nlm.nih.gov/genomes/all/GCF/000/003/605/GCF_000003605.2_Skow_1.1/GCF_000003605.2_Skow_1.1_protein.faa.gz | Skow_1.1 | 20170119 |
| 7460 | Apis melifera | genome | ftp://ftp.ensemblgenomes.org/pub/metazoa/release-34/fasta/apis_mellifera/pep/Apis_mellifera.GCA_000002195.1.pep.all.fa.gz | Amel_4.5 | 20170119 |
| 7091 | Bombyx mori | genome | ftp://ftp.ensemblgenomes.org/pub/metazoa/release-34/fasta/bombyx_mori/pep/Bombyx_mori.GCA_000151625.1.pep.all.fa.gz | ASM15162v1 | 20170119 |
| 9258 | Ornithorhynchus anatinus | genome | ftp://ftp.ensembl.org/pub/release-87/fasta/ornithorhynchus_anatinus/pep/Ornithorhynchus_anatinus.OANA5.pep.all.fa.gz | OANA5 | 20170119 |
| 9606 | Homo sapiens | genome | ftp://ftp.ensembl.org/pub/release-87/fasta/homo_sapiens/pep/ | GRCH38.p7 | 20170119 |
| 6850 | Limulus polyphemus | genome | ftp://ftp.ncbi.nlm.nih.gov/genomes/all/GCF/000/517/525/GCF_000517525.1_Limulus_polyphemus-2.1.2/GCF_000517525.1_Limulus_polyphemus-2.1.2_protein.faa.gz | Limulus polyphemus-2.1.2 | 20170119 |
| 1819745 | Intoshia linei | genome | ftp://ftp.ncbi.nlm.nih.gov/genomes/all/GCA/001/642/005/GCA_001642005.1_IntLin1.0/GCA_001642005.1_IntLin1.0_protein.faa.gz | IntLin_1.0 | 20170119 |
| 6279 | Brugia malayi | genome | ftp://ftp.ensemblgenomes.org/pub/metazoa/release-34/fasta/brugia_malayi/pep/Brugia_malayi.B_malayi-3.1.pep.all.fa.gz | B_malayi-3.1 | 20170119 |
| 6239 | Caenorhabditis elegans | genome | ftp://ftp.ensemblgenomes.org/pub/metazoa/release-34/fasta/caenorhabditis_elegans/pep/Caenorhabditis_elegans.WBcel235.pep.all.fa.gz | Wcel235 | 20170119 |
| 6334 | Trichinella spiralis | genome | ftp://ftp.ncbi.nlm.nih.gov/genomes/all/GCF/000/181/795/GCF_000181795.1_Trichinella_spiralis-3.7.1/GCF_000181795.1_Trichinella_spiralis-3.7.1_protein.faa.gz | Trichinella spiralis-3.7.1 | 20170119 |
| 121225 | Pediculus humanus | genome | ftp://ftp.ensemblgenomes.org/pub/metazoa/release-34/fasta/pediculus_humanus/pep/Pediculus_humanus.PhumU2.pep.all.fa.gz | PhumU2 | 20170119 |
| 10228 | Trichoplax adhaerens | genome | ftp://ftp.ensemblgenomes.org/pub/metazoa/release-34/fasta/trichoplax_adhaerens/pep/Trichoplax_adhaerens.ASM15027v1.pep.all.fa.gz | ASM15027v1 | 20170119 |
| 60517 | Taenia asiatica | genome | ftp://ftp.ebi.ac.uk/pub/databases/wormbase/parasite/releases/WBPS8/species/taenia_asiatica/PRJEB532/taenia_asiatica.PRJEB532.WBPS8.protein.fa.gz | T_asiatica_South_Korea_v1_0_4 | 20170119 |
| 79327 | Schmidtea mediterranea | genome | ftp://ftp.ebi.ac.uk/pub/databases/wormbase/parasite/releases/WBPS8/species/schmidtea_mediterranea/PRJNA12585/schmidtea_mediterranea.PRJNA12585.WBPS8.protein.fa.gz or http://smedgd.stowers.org/files/SmedSxl_genome_v4.0.all.maker.proteins.fasta.gz | SmedGD_v1.3 and SmedGD_v4.0 | 20170119 |
| 6183 | Schistosoma mansoni | genome | ftp://ftp.ebi.ac.uk/pub/databases/wormbase/parasite/releases/WBPS8/species/schistosoma_mansoni/PRJEA36577/schistosoma_mansoni.PRJEA36577.WBPS8.protein.fa.gz | ASM23792v2 | 20170119 |
| 400682 | Amphimedon queenslandica | genome | http://amphimedon.qcloud.qcif.edu.au/downloads.html | Aqu2.1 | 20170119 |
| 13735 | Pelodiscus sinensis | genome | ftp://ftp.ensembl.org/pub/release-87/fasta/pelodiscus_sinensis/pep/Pelodiscus_sinensis.PelSin_1.0.pep.all.fa.gz | PelSin_1.0 | 20170119 |
| 104782 | Adineta vaga | genome | http://www.genoscope.cns.fr/adineta/data/Adineta_vaga.v2.pep.fa.gz | Adineta_vaga.v2 | 20170119 |
| 947166 | Ramazzottius varieornatus | genome | ftp://ftp.ncbi.nlm.nih.gov/genomes/all/GCA/001/949/185/GCA_001949185.1_Rvar_4.0/GCA_001949185.1_Rvar_4.0_protein.faa.gz | rvar_4.0 | 20170119 |
| 34765 | Oikopleura dioica | genome | ftp://ftp.ncbi.nlm.nih.gov/genomes/all/GCA/000/209/535/GCA_000209535.1_ASM20953v1/GCA_000209535.1_ASM20953v1_protein.faa.gz | Oikopleura_peptides_reference_v1.0 | 20170119 |
| 7719 | Ciona intestinalis | genome | ftp://ftp.ensembl.org/pub/release-87/fasta/ciona_intestinalis/pep/Ciona_intestinalis.KH.pep.all.fa.gz | KH | 20170119 |
| 37621 | Priapulus caudatus | genome | ftp://ftp.ncbi.nlm.nih.gov/genomes/all/GCF/000/485/595/GCF_000485595.1_Priapulus_caudatus-5.0.1/GCF_000485595.1_Priapulus_caudatus-5.0.1_protein.faa.gz | Priapulus caudatus-5.0.1 | 20170119 |
| 81824 | Monosiga brevicollis | genome | ftp://ftp.ensemblgenomes.org/pub/protists/release-34/fasta/protists_choanoflagellida1_collection/monosiga_brevicollis_mx1/pep/Monosiga_brevicollis_mx1.V1.0.pep.all.fa.gz | MonBr V1.0, | 20170120 |
| 946362 | Salpingoeca rosetta | genome | ftp://ftp.ensemblgenomes.org/pub/protists/release-34/fasta/protists_choanoflagellida1_collection/salpingoeca_rosetta/pep/Salpingoeca_rosetta.Proterospongia_sp_ATCC50818.pep.all.fa.gz | Proterospongia_sp_ATCC50818 | 20170120 |
| 595528 | Capsaspora owczarzaki 30864 | genome | https://figshare.com/articles/dataset/Genome_-_Capsaspora_owczarzaki_v3_/4123158 | Capsaspora owczarzaki (v3) | 20220920 |
| 5341 | Agaricus bisporus | genome | ftp://ftp.ensemblgenomes.org/pub/fungi/release-34/fasta/fungi_basidiomycota1_collection/agaricus_bisporus_var_bisporus_h97/pep/Agaricus_bisporus_var_bisporus_h97.Agabi_varbisH97_2.pep.all.fa.gz | Agabi_varbisH97_2 | 20170120 |
| 5207 | Cryptococcus neoformans | genome | ftp://ftp.ensemblgenomes.org/pub/fungi/release-34/fasta/fungi_basidiomycota1_collection/cryptococcus_neoformans_var_grubii_h99/pep/Cryptococcus_neoformans_var_grubii_h99.CNA3.pep.all.fa.gz | CNA3 | 20170120 |
| 209285 | Chaetomium thermophilum | genome | http://ct.bork.embl.de/downloads.html | C_thermophilum.annotation.v2.4.tar.gz | 20181107 |
| 1305733 | Rhodotorula sp | genome | ftp://ftp.ensemblgenomes.org/pub/fungi/release-34/fasta/fungi_basidiomycota1_collection/rhodotorula_sp_jg_1b/pep/Rhodotorula_sp_jg_1b.Rhosp1.pep.all.fa.gz | Rhosp1 | 20170120 |
| 28583 | Allomyces macrogynus | genome | ftp://ftp.ensemblgenomes.org/pub/fungi/release-34/fasta/fungi_blastocladiomycota1_collection/allomyces_macrogynus_atcc_38327/pep/Allomyces_macrogynus_atcc_38327.A_macrogynus_V3.pep.all.fa.gz | A_macrogynus_V3 | 20170120 |
| 109876 | Catenaria anguillulae | genome | http://genome.jgi.doe.gov/Catan2/download/Catan2_all_proteins_20160412.aa.fasta.gz | Catan2 | 20170120 |
| 109871 | Batrachochytrium dendrobatidis | genome | ftp://ftp.ensemblgenomes.org/pub/fungi/release-34/fasta/fungi_chytridiomycota1_collection/batrachochytrium_dendrobatidis_jam81/pep/Batrachochytrium_dendrobatidis_jam81.v1.0.pep.all.fa.gz | V1.0 | 20170120 |
| 645134 | Spizellomyces punctatus DAOM | genome | ftp://ftp.ensemblgenomes.org/pub/fungi/release-34/fasta/fungi_chytridiomycota1_collection/spizellomyces_punctatus_daom_br117/pep/Spizellomyces_punctatus_daom_br117.S_punctatus_V1.pep.all.fa.gz | S_punctatis_V1 | 20170120 |
| 423460 | Basidiobolus meristosporus | genome | http://genome.jgi.doe.gov/Basme2finSC/download/Basme2finSC_GeneCatalog_proteins_20150701.aa.fasta.gz | BasmefinSC | 20170120 |
| 796925 | Condiobolus coronatus | genome | ftp://ftp.ensemblgenomes.org/pub/fungi/release-34/fasta/fungi_entomophthoromycota1_collection/conidiobolus_coronatus_nrrl_28638/pep/Conidiobolus_coronatus_nrrl_28638.Conidiobolus_coronatus_NRRL28638.pep.all.fa.gz | Conidiobolus coronatus NRRL28638 | 20170120 |
| 588596 | Rhizophagus irregularis | genome | ftp://ftp.ensemblgenomes.org/pub/fungi/release-34/fasta/fungi_glomeromycota1_collection/rhizophagus_irregularis_daom_181602/pep/Rhizophagus_irregularis_daom_181602.Gloin1.pep.all.fa.gz | Gloin1 | 20170120 |
| 61392 | Coemansia reversa | genome | genome.jgi.doe.gov/Coere1/download/Coere1_GeneCatalog_proteins_20110909.aa.fasta.gz | Coere1 | 20170120 |
| 304332 | Ramicandelaber brevisporus | genome | http://genome.jgi.doe.gov/Rambr1/download/Rambr1_GeneCatalog_proteins_20140929.aa.fasta.gz | Rambr1 | 20170120 |
| 1485682 | Mitosporidium daphniae | genome | ftp://ftp.ensemblgenomes.org/pub/fungi/release-34/fasta/fungi_microsporidia1_collection/mitosporidium_daphniae/pep/Mitosporidium_daphniae.UGP1.0.pep.all.fa.gz | UGP1.0 | 20170125 |
| 58839 | Encephalitozoon intestinalis | genome | ftp://ftp.ensemblgenomes.org/pub/fungi/release-34/fasta/fungi_microsporidia1_collection/encephalitozoon_intestinalis_atcc_50506/pep/Encephalitozoon_intestinalis_atcc_50506.ASM14646v1.pep.all.fa.gz | ASM14646v1 | 20170125 |
| 78898 | Mortierella verticillata | genome | http://genome.jgi.doe.gov/Morve1/download/Morve1_GeneCatalog_proteins_20161024.aa.fasta.gz | Morve1 | 20170120 |
| 310910 | Mortierella elongata | genome | http://genome.jgi.doe.gov/Morel2/download/Morel2_GeneCatalog_proteins_20151120.aa.fasta.gz | Morel2 | 20170120 |
| 36080 | Mucor circinelloides | genome | http://genome.jgi.doe.gov/Mucci2/download/Mucor_circinelloides_v2_filtered_proteins.fasta.gz | Muccl2 | 20170120 |
| 4837 | Phycomyces blakesleeanus | genome | http://genome.jgi.doe.gov/Phybl2/download/Phycomyces_blakesleeanus_v2_filtered_proteins.fasta.gz | Phybl2 | 20170120 |
| 1577477 | Piromyces sp finn | genome | http://genome.jgi.doe.gov/Pirfi3/download/Pirfi3_GeneCatalog_proteins_20160330.aa.fasta.gz | Pirfi3 | 20170120 |
| 1550276 | Neocallimastix sp | genome | http://genome.jgi.doe.gov/Neosp1/download/Neosp1_GeneCatalog_proteins_20160330.aa.fasta.gz | Neosp1 | 20170120 |
| 5141 | Neurospora crassa | genome | ftp://ftp.ensemblgenomes.org/pub/fungi/release-34/fasta/fungi_ascomycota2_collection/neurospora_crassa_gca_000786625/pep/Neurospora_crassa_gca_000786625.Neucr_trp3_1.pep.all.fa.gz | Neucr_trp3_1 | 20170208 |
| 39416 | Tuber melanosporum | genome | ftp://ftp.ensemblgenomes.org/pub/fungi/release-34/fasta/tuber_melanosporum/pep/Tuber_melanosporum.ASM15164v1.pep.all.fa.gz | ASM15164v1 | 20170208 |
| 242477 | Melampsora laricis | genome | http://genome.jgi.doe.gov/Mellp2_3/download/Mellp2_3_GeneCatalog_proteins_20151130.aa.fasta.gz | mellp2_3 | 20170120 |
| 4932 | Saccharomyces cerevisiae | genome | ftp://ftp.ensemblgenomes.org/pub/fungi/release-34/fasta/saccharomyces_cerevisiae/pep/Saccharomyces_cerevisiae.R64-1-1.pep.all.fa.gz | R64-1-1 | 20170208 |
| 28985 | Kluyveromyces lactis | genome | ftp://ftp.ensemblgenomes.org/pub/fungi/release-34/fasta/fungi_ascomycota1_collection/kluyveromyces_lactis/pep/Kluyveromyces_lactis.ASM251v1.pep.all.fa.gz | ASM251v1 | 20170208 |
| 4952 | Yarrowia lipolytica | genome | ftp://ftp.ncbi.nlm.nih.gov/genomes/all/GCA/001/761/485/GCA_001761485.1_ASM176148v1/GCA_001761485.1_ASM176148v1_protein.faa.gz | ASM176148v1 | 20170208 |
| 5606 | Saitoella complicata | genome | ftp://ftp.ensemblgenomes.org/pub/fungi/release-34/fasta/fungi_ascomycota2_collection/saitoella_complicata_nrrl_y_17804/pep/Saitoella_complicata_nrrl_y_17804.Scomplicata_3.0.pep.all.fa.gz | Scomplicata_3.0 | 20170120 |
| 4896 | Schizosaccharomyces pombe | genome | ftp://ftp.ensemblgenomes.org/pub/fungi/release-34/fasta/schizosaccharomyces_pombe/pep/Schizosaccharomyces_pombe.ASM294v2.pep.all.fa.gz | ASM294v2 | 20170120 |
| 5270 | Ustilago maydis | genome | ftp://ftp.ensemblgenomes.org/pub/fungi/release-34/fasta/ustilago_maydis/pep/Ustilago_maydis.Umaydis521_2.0.pep.all.fa.gz | Umaydis521_2.0 | 20170120 |
| 1851185 | Syncephalis plumigaleata | genome | http://genome.jgi.doe.gov/Synplu1/download/Synplu1_primary_alleles_proteins_20160908.aa.fasta.gz | synplu1 | 20170120 |
| 281847 | Rozella allomycis | genome | ftp://ftp.ensemblgenomes.org/pub/fungi/release-34/fasta/fungi_rozellomycota1_collection/rozella_allomycis_csf55/pep/Rozella_allomycis_csf55.Rozella_k41_t100.pep.all.fa.gz | Rozella_k41_t100 | 20170120 |
| 749232 | Abeoforma whisleri | genome | https://figshare.com/articles/Genome_-_Abeoforma_whisleri_/5426458 |  | 20181111 |
| 1932427 | Chromosphaera perkinsii | genome | https://figshare.com/articles/Genome_-_Chromosphaera_perkinsii/5426494 | | 20181111 |
| 39843 | Ichthyophonus hoferi | genome | https://figshare.com/articles/Genome_-_Ichthyophonus_hoferi/5426488 |  | 20181111 |
| 749231 | Pirum gemmata | genome | https://figshare.com/articles/Genome_-_Pirum_gemmata/5426506 |  | 20181111 |
| 72019 | Sphaeroforma arctica | genome | https://figshare.com/articles/dataset/Sphaeroforma_arctica_transcriptome/8299529 , , 20220920 | Sarc4 | 20220920 |
| 470921 | Creolimax fragrantissima | genome | https://figshare.com/articles/Creolimax_fragrantissima_genome_data/1403592 | Creollimax_fragrantissima | 20170120 |
| 1553916 | Nuclearia sp | transcriptome | https://figshare.com/articles/Nuclearia_sp_ATCC_50694_-_Transcriptome/3898485 | Nuclearia_a_unigene | 20170120 |
| 691883 | Fonticula alba | genome | ftp://ftp.ncbi.nlm.nih.gov/genomes/all/GCA/000/388/065/GCA_000388065.2_Font_alba_ATCC_38817_V2/GCA_000388065.2_Font_alba_ATCC_38817_V2_protein.faa.gz | Font_alba_ATCC_38817_V2 | 20170120 |
| 2717461 | Colponemidia sp Colp-10 | transcriptome | https://sra-download.ncbi.nlm.nih.gov/traces/wgs01/wgs_aux/GI/LJ/GILJ01/GILJ01.1.fsa_nt.gz | GILJ01000000 | 20210527 |
| 2717462 | Colponemidia sp Colp-15 | transcriptome | https://sra-download.ncbi.nlm.nih.gov/traces/wgs01/wgs_aux/GI/LK/GILK01/GILK01.1.fsa_nt.gz | GILK01000000 | 20210527 |
| 5833 | Plasmodium falciparum | genome | ftp://ftp.ensemblgenomes.org/pub/release-34/protists/fasta/plasmodium_falciparum/pep/ | ASM276v1 | 20161212 |
| 5866 | Babesia bigemina | genome | ftp://ftp.ensemblgenomes.org/pub/release-34/protists/fasta/protists_alveolata1_collection/babesia_bigemina/pep/ | Bbig001 | 20161212 |
| 5874 | Theileria annulata | genome | ftp://ftp.ensemblgenomes.org/pub/release-34/protists/fasta/protists_alveolata1_collection/theileria_annulata/pep/ | ASM322v1 | 20161212 |
| 110365 | Gregarina niphandrodes | genome | ftp://ftp.ensemblgenomes.org/pub/release-34/protists/fasta/protists_alveolata1_collection/gregarina_niphandrodes/pep/ | GNI3 | 20161212 |
| 5808 | Cryptosporidium muris | genome | ftp://ftp.ensemblgenomes.org/pub/release-34/protists/fasta/protists_alveolata1_collection/cryptosporidium_muris_rn66/pep/ | JCVI-cmg-v1.0 | 20161212 |
| 5807 | Cryptosporidium parvum | genome | ftp://ftp.ensemblgenomes.org/pub/release-34/protists/fasta/protists_alveolata1_collection/cryptosporidium_parvum_iowa_ii/pep/ | ASM16534v1 | 20161212 |
| 5811 | Toxoplasma gondii | genome | ftp://ftp.ensemblgenomes.org/pub/release-34/protists/fasta/toxoplasma_gondii/pep/ | ToxoDB-7.1 | 20161212 |
| 5801 | Eimeria acervulina | genome | ftp://ftp.ensemblgenomes.org/pub/release-34/protists/fasta/protists_alveolata1_collection/eimeria_acervulina/pep/ | EAH001 | 20161212 |
| 505693 | Chromera velia | genome | http://cryptodb.org/common/downloads/Current_Release/CveliaCCMP2878/fasta/data/ | ? | 20170208 |
| 1169539 | Vitrella brassicaformis | genome | ftp://ftp.ensemblgenomes.org/pub/release-34/protists/fasta/protists_alveolata1_collection/vitrella_brassicaformis_ccmp3155/pep/ | Vbrassicaformis | 20161212 |
| 5888 | Paramecium tetraurelia | genome | ftp://ftp.ensemblgenomes.org/pub/release-34/protists/fasta/paramecium_tetraurelia/pep/ | GCA_000165425.1 | 20161212 |
| 5911 | Tetrahymena thermophila | genome | ftp://ftp.ensemblgenomes.org/pub/release-34/protists/fasta/tetrahymena_thermophila/pep/ | JCVI-TTA1-2.2 | 20161212 |
| 5932 | Ichthyophthirius multifiliis | genome | ftp://ftp.ensemblgenomes.org/pub/release-34/protists/fasta/protists_alveolata1_collection/ichthyophthirius_multifiliis/pep/ | JCVI-IMG1-V.1 | 20161212 |
| 266149 | Pseudocohnilembus persalinus | genome | ftp://ftp.ensemblgenomes.org/pub/release-34/protists/fasta/protists_alveolata1_collection/pseudocohnilembus_persalinus/pep/ | ASM144751v1 | 20161212 |
| 5949 | Stylonychia lemnae | genome | ftp://ftp.ensemblgenomes.org/pub/release-34/protists/fasta/protists_alveolata1_collection/stylonychia_lemnae/pep/ | Stylonychia_lemnae_asm_v1.0 | 20161212 |
| 1172189 | Oxytricha trifallax | genome | ftp://ftp.ensemblgenomes.org/pub/release-34/protists/fasta/protists_alveolata1_collection/oxytricha_trifallax_gca_000295675/ | GCA_00295675 | 20161212 |
| 5963 | Stentor coeruleus | genome | https://www.ncbi.nlm.nih.gov/assembly/GCA_001970955.1/ | ASM197095v1 \| S_coeruleus_Nov216 | 20170210 |
| 31276 | Perkinsus marinus | genome | ftp://ftp.ensemblgenomes.org/pub/release-34/protists/fasta/protists_alveolata1_collection/perkinsus_marinus_atcc_50983/pep/ | JCVI_PMG_1.0 | 20161212 |
| 2951 | Symbiodinium microadriaticum | genome | ftp://ftp.ncbi.nlm.nih.gov/genomes/all/GCA/001/939/145/GCA_001939145.1_ASM193914v1/GCA_001939145.1_ASM193914v1_protein.faa.gz | ASM193914v1 | 20170207 |
| 1202447 | Symbiodinium minutum | genome | http://marinegenomics.oist.jp/symb/viewer/download?project_id=21 (Assembly V1.0, symbB.v1.2.augustus.prot.fa.gz) | Symbiodinium minutum ver. symb_aug_v1.120123 | 20161212 |
| 37360 | Plasmodiophora brassicae | genome | ftp://ftp.ensemblgenomes.org/pub/release-34/protists/fasta/protists_rhizaria1_collection/plasmodiophora_brassicae/pep/ | pbe3.h15 | 20161212 |
| 46433 | Reticulomyxa filosa | genome | ftp://ftp.ensemblgenomes.org/pub/release-34/protists/fasta/protists_rhizaria1_collection/reticulomyxa_filosa/pep/ | Reti_assembly1.0 | 20161212 |
| 227086 | Bigelowiella natans | transcriptome | https://figshare.com/articles/Marine_Microbial_Eukaryotic_Transcriptome_Sequencing_Project_re-assemblies/3840153/3 | version 3 | 20181113 |
| 552665 | Bigelowiella longifila | transcriptome | https://figshare.com/articles/Marine_Microbial_Eukaryotic_Transcriptome_Sequencing_Project_re-assemblies/3840153/3 | version 3 | 20181113 |
| 29199 | Chlorarachnion reptans | transcriptome | https://figshare.com/articles/Marine_Microbial_Eukaryotic_Transcriptome_Sequencing_Project_re-assemblies/3840153/3 | version 3 | 20181113 |
| 2850 | Phaeodactylum tricornutum | genome | ftp://ftp.ensemblgenomes.org/pub/release-34/protists/fasta/phaeodactylum_tricornutum/pep/ | ASM15095v2 | 20161212 |
| 35128 | Thalassiosira pseudonana | genome | ftp://ftp.ensemblgenomes.org/pub/release-34/protists/fasta/thalassiosira_pseudonana/pep/ | ASM14940v2 | 20161212 |
| 186039 | Fragilariopsis cylindrus | genome | http://genome.jgi.doe.gov/pages/dynamicOrganismDownload.jsf?organism=Fracy1 (Fracy1_GeneModels_FilteredModels1_aa.fasta.gz) | v1.0 | 20170105 |
| 72520 | Nannochloropsis gaditana | genome | http://www.nannochloropsis.org/page/ftp | CCMP526 | 20170327 |
| 87111 | Aplanochytrium kerguelense | genome | http://genome.jgi.doe.gov/pages/dynamicOrganismDownload.jsf?organism=Aplke1 (Aplke1_GeneCatalog_proteins_20121220.aa.fasta.gz) | V1.0 | 20161212 |
| 4773 | Schizochytrium aggregatum | genome | http://genome.jgi.doe.gov/pages/dynamicOrganismDownload.jsf?organism=Schag1 (Schag1_GeneCatalog_proteins_20121220.aa.fasta.gz) | V1.0 | 20161212 |
| 87102 | Aurantiochytrium limacinum | genome | http://genome.jgi.doe.gov/pages/dynamicOrganismDownload.jsf?organism=Aurli1 (Aurli1_GeneCatalog_proteins_20120618.aa.fasta.gz) | V1.0 | 20161212 |
| 4787 | Phytophthora infestans | genome | ftp://ftp.ensemblgenomes.org/pub/release-34/protists/fasta/phytophthora_infestans/pep/ | ASM14294v1 | 20161212 |
| 123356 | Hyaloperonospora parasitica | genome | ftp://ftp.ensemblgenomes.org/pub/release-34/protists/fasta/hyaloperonospora_arabidopsidis/pep/ | HyaAraEmoy2_2.0 | 20161212 |
| 4781 | Plasmopara halstedii | genome | ftp://ftp.ensemblgenomes.org/pub/release-34/protists/fasta/protists_stramenopiles1_collection/plasmopara_halstedii/pep/ | | 20161212 |
| 2052682 | Pythium ultimum | genome | ftp://ftp.ensemblgenomes.org/pub/release-34/protists/fasta/pythium_ultimum/pep/ | | 20161212 |
| 653948 | Albugo laibachii | genome | ftp://ftp.ensemblgenomes.org/pub/release-34/protists/fasta/albugo_laibachii/pep/ | ENA 1 | 20161212 |
| 112090 | Aphanomyces astaci | genome | ftp://ftp.ensemblgenomes.org/pub/release-34/protists/fasta/protists_stramenopiles1_collection/aphanomyces_astaci/pep/ | Apha_asta_APO3_V1 | 20161212 |
| 101203 | Saprolegnia parasitica | genome | ftp://ftp.ensemblgenomes.org/pub/release-34/protists/fasta/protists_stramenopiles1_collection/saprolegnia_parasitica_cbs_223_65/pep/ | ASM15154v2 | 20161212 |
| 12968 | Blastocystis hominis | genome | ftp://ftp.ensemblgenomes.org/pub/release-34/protists/fasta/protists_stramenopiles1_collection/blastocystis_hominis/pep/ | ASM15166v1 | 20161212 |
| 44056 | Aureococcus anophagefferens | genome | ftp://ftp.ensemblgenomes.org/pub/release-34/protists/fasta/protists_stramenopiles1_collection/aureococcus_anophagefferens/pep/ | v 1.0 | 20161212 |
| 2880 | Ectocarpus siliculosus | genome | https://bioinformatics.psb.ugent.be/gdb/ectocarpus/ (EctsiV2_prot_LATEST.tfa.gz) | EctsiV2 | 20161212 |
| 309737 | Cladosiphon okamuranus | genome | http://marinegenomics.oist.jp/algae/viewer/download?project_id=53 (downloaded file: 160208_2k_oki_prot.fa.gz) | Assembly v1.0 | 20161212 |
| 529818 | Thecamonas trahens | genome | ftp://ftp.ensemblgenomes.org/pub/protists/release-34/fasta/protists_apusozoa1_collection/thecamonas_trahens_atcc_50062/pep/Thecamonas_trahens_atcc_50062.TheTra_May2010.pep.all.fa.gz | TheTra_May2010 | 20170120 |
| 221724 | Seculamonas sp ecuadoriensis | EST | https://www.ncbi.nlm.nih.gov/nuccore?LinkName=biosample_nuccore&from_uid=150550 | LIBEST_019958 | 20210527 |
| 392300 | Histiona aroides | EST | https://www.ncbi.nlm.nih.gov/nuccore?LinkName=biosample_nuccore&from_uid=150562 | LIBEST_019970 | 20210527 |
| 221721 | Jakoba bahamiensis | EST | https://www.ncbi.nlm.nih.gov/nuccore?LinkName=biosample_nuccore&from_uid=150510 | LIBEST_019913 | 20210527 |
| 143017 | Jakoba libera | EST | https://www.ncbi.nlm.nih.gov/nuccore?LinkName=biosample_nuccore&from_uid=150512 | LIBEST_019915 | 20210527 |
| 48483 | Reclinomonas americana | EST | https://www.ncbi.nlm.nih.gov/nuccore?LinkName=biosample_nuccore&from_uid=150540,https://www.ncbi.nlm.nih.gov/nuccore?LinkName=biosample_nuccore&from_uid=150541,https://www.ncbi.nlm.nih.gov/nuccore?LinkName=biosample_nuccore&from_uid=150542,https://www.ncbi.nlm.nih.gov/nuccore?LinkName=biosample_nuccore&from_uid=150543,https://www.ncbi.nlm.nih.gov/nuccore?LinkName=biosample_nuccore&from_uid=150544,https://www.ncbi.nlm.nih.gov/nuccore?LinkName=biosample_nuccore&from_uid=150545,https://www.ncbi.nlm.nih.gov/nuccore?LinkName=biosample_nuccore&from_uid=150546 | LIBEST_019948,LIBEST_019949,LIBEST_019950,LIBEST_019951,LIBEST_019952,LIBEST_019953,LIBEST_019954 | 20210527 |
| 505711 | Andalucia godoyi | genome | https://megasun.bch.umontreal.ca/Andalucia_godoyi/Andalucia_godoyi_proteome.faa | Andalucia_godoyi_proteome | 20210527 |

**Supplementary text**

*Contamination is not evident in our dataset*

To exclude contamination in our dataset we used two methods. First, we searched each sequence against the NR dataset using BLASTP. We set the contamination threshold as such that when a hit is above 80% sequence identity outside of the major eukaryotic group or 95% within the major eukaryotic group it would be identified as contaminated. Second, we manually inspected the topology of the gene trees. Genes would be identified as contamination when there was a short branch, and the sister was not part of the major eukaryotic group. We did not identify any contamination using both methods.
